# Preanalytical (Mis)Handling of Plasma Investigated by ^1^H NMR Metabolomics

**DOI:** 10.1021/acsomega.4c08215

**Published:** 2024-11-27

**Authors:** Daniel Malmodin, Anders Bay Nord, Huma Zafar, Linda Paulson, B. Göran Karlsson, Åsa Torinsson Naluai

**Affiliations:** † Swedish NMR Centre at the University of Gothenburg, SE-405 30 Gothenburg, Sweden; ‡ Biobank Väst, SE-413 45 Gothenburg, Sweden; § Biobank Core Facility, SE-405 30 Gothenburg, Sweden; ∥ Institute of Biomedicine, Sahlgrenska Academy, University of Gothenburg, SE-405 30 Gothenburg, Sweden; ⊥ National Bioinformatics Infrastructure Sweden (NBIS), University of Gothenburg, SE-405 30 Gothenburg, Sweden

## Abstract

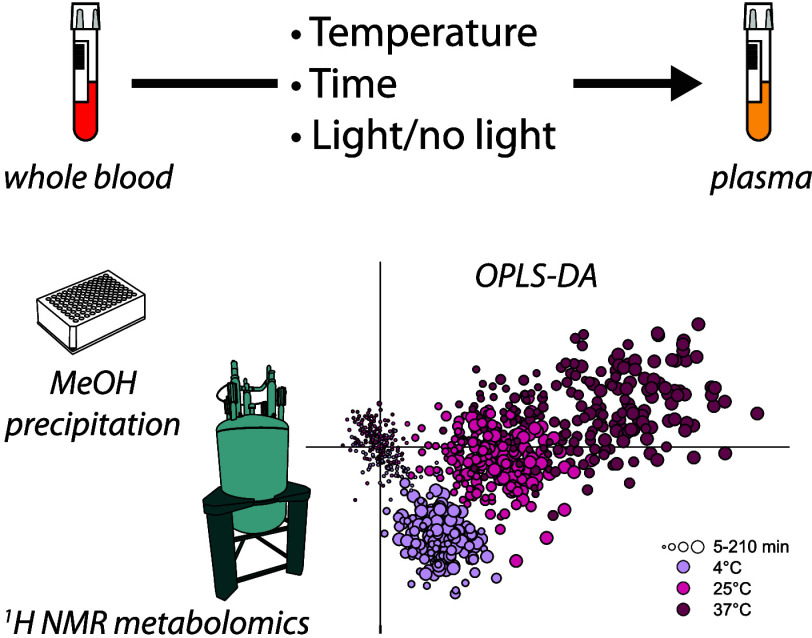

The preanalytical handling of plasma, how it is drawn, processed, and stored, influences its composition. Samples in biobanks often lack this information and, consequently, important information about their quality. Especially metabolite concentrations are affected by preanalytical handling, making conclusions from metabolomics studies particularly sensitive to misinterpretations. The perturbed metabolite profile, however, also offers an attractive choice for assessing the preanalytical history from the measured data. Here we show that it is possible using Orthogonal Projections to Latent Structures Discriminative Analysis to divide plasma NMR data into a multivariate “original sample space” suitable for further less biased metabolomics analysis and an orthogonal “preanalytical handling space” describing the changes occurring from preanalytical mishandling. Apart from confirming established preanalytical effects on metabolite levels, e.g., the consequent changes in glucose, lactate, ornithine, and pyruvate, the sample preparation protocol involved methanol precipitation which allowed the observation of reversible changes in short-chain fatty acid concentrations as a function of temperature.

## Introduction

Proton nuclear magnetic resonance spectroscopy (^1^H NMR) offers some unique possibilities compared to the expanding universe of mass spectrometry metabolomics applications, namely, the facile, directly quantitative, and highly reproducible acquisition of metabolite data. Finding and characterizing metabolomic biomarkers important for human disease often require large sample cohorts, more so if an underlying confounding variability exists from both preanalytical and postanalytical processing. In an ideal case, the variability in the data set should derive from the original sample content rather than the sample handling since even in such a situation, a lot of observed variability is present naturally (such as biological or technical variability), unrelated to the research question at hand.

Knowledge about the quality of a stored biofluid sample in terms of how representative it is of the biofluid at the actual time of sampling is crucial but is often overlooked. The surge in biobanking efforts and increase in analytical requests on biobanked material requires establishing proper preanalytical sample standard operating procedures assuring sample quality, not the least because a lot of the variability in clinical chemistry comes from errors in the handling process.^1,2^ The analytical method to be used in a given study is impacted differently depending on the preanalytical procedures that were used. Arguably, metabolomics is the technique most sensitive to differences in preanalytical handling as minute changes in metabolite levels can be detected and as such can be the consequence of slight sampling procedure variation. This was highlighted in recent work where samples from multiple biobanks were analyzed with NMR and the results showed that the differences between the biobanks (i.e., the preanalytical handling) were greater than the interindividual variation.^3^ Previous studies to assess the quality of biobank samples have focused on different aspects of sample quality, such as preanalytical time to freezer,^4−7^ centrifugation speed,^8^ and/or temperature,^4−7^ as well as the effect of freeze–thaw cycles^7,9^ and storage at different freezer temperatures.^10−12^ For recent reviews on the effects of preanalytical handling on metabolite stability and recommendations, see Stevens et al.,^13^ Kirwan et al.,^14^ and Gonzalez-Dominguez et al.^15^ There is also an established preanalytical sample preparation standard, ISO 23118:2021.

NMR has previously been suggested as a good choice for quality assessment of biobank samples,^16^ and here, we report the effects of preanalytical temperature, time to centrifugation, and light/no light on the ^1^H NMR metabolome of plasma samples collected in K_2_-EDTA tubes that were subjected to methanol precipitation before data acquisition. An LC–MS-based study of the same plasma raw material has been published.^17^

Orthogonal Projections to Latent Structures^18^ is a popular and excellent multivariate data processing method that removes systematic variation in a data set orthogonal to define property variables. The remaining data are divided between a PLS model describing systematic variation with the property variables and unstructured data often interpreted as noise. The property variables are either continuous or discrete. The latter can also be used to describe group belongings and is then called Orthogonal projections to latent structures—Discriminant Analysis.^19,20^ Almost always, the aim of orthogonal partial least-squares-discriminant analysis (OPLS-DA) is to explicitly use the correlations between the systematic variations in the data and the property variables, where success in terms of separating groups is described by the value *Q*^2^. Using OPLS-DA only for filtering systematic orthogonal information is much less common.

## Objectives

The present work aims to show the utility of describing the effects of preanalytical handling (varying temperature, time to centrifugation, and light/dark conditions) on drawn blood samples by dividing the data into a multivariate “original sample space” and an orthogonal “preanalytical handling space.” This is demonstrated on deproteinized plasma ^1^H NMR spectra.

## Methods

### Subjects

Blood samples were collected from 28 healthy volunteers, 19 females, and 9 males, with ages ranging from 20 to 76 years old. All participants provided informed consent prior to enrolling in the study, which was carried out in accordance with the Declaration of Helsinki and Swedish Biobank legislation. All volunteers were asked to participate twice, and 13 did, giving in total 41 original samples. At the first occasion, they were asked to give a 4 h fasting sample prior to blood withdrawal, and at the second visit, they were asked to be in a nonfasting state. The study was approved by the regional ethics board in Gothenburg, reference number 054-15.

### Plasma Sampling and Preanalytical Treatment

Unless otherwise stated, chemicals were acquired from Merck. Blood samples were collected in K_2_-EDTA 15.8 mg collection tubes including a gel separator (Vacutainer, Becton Dickinson). Every participant’s blood ideally filled 24 tubes at each occasion, in principle giving 984 samples, but for various reasons, only 952 were collected for analyses. One of the samples was unusable, meaning that the main study analyses comprised 951 samples. The tubes were stored at three different temperatures 4, 25, or 37 °C, during four different time periods of approximately 0 h, 1 h 30 min, 2 h 30 min, or 3 h 30 min. Samples were kept either in darkness (tube covered in foil) or exposed to normal indoor room lighting before centrifugation at 2000*g* for 10 min. Samples stored at 4 °C were centrifuged at 4 °C and samples at 25 or 37 °C were centrifuged at RT. Samples were collected and pipetted into 96-well plates (REMP, Brooks Life Sciences) for cold storage (−80 °C). Additionally, eight EDTA tubes were taken at four separate sampling occasions from one participant. At each occasion, tubes were put at 4 or 25 °C for approximately 10, 30, or 60 min, or at 4 °C for approximately 30 min followed by 30 min at 25 °C or at 25 °C for approximately 30 min followed by 30 min at 4 °C, followed by centrifugation at either 4 °C or RT depending on what the last temperature incubation was.

### Sample Preprocessing

Plasma samples in a REMP plate were thawed at 4 °C overnight. 150 μL of plasma per well was transferred to 96-well deepwell plates (DWP; Porvair cat. no. 219009, 2 mL of polypropylene, square wells) using a Bravo 96-channel liquid handler (Agilent Technologies). Methanol precipitation was performed essentially as described in Nagana Gowda et al.^21^ Briefly, a Bravo liquid handler was used to mix 150 μL of thawed serum with 750 μL of cold (−20 °C) methanol in the 96-well deepwell plate. The Bravo robot operated with 250 μL filter tips, necessitating several pipetting steps for the methanol addition. The plate was sealed with a piercable sealing cap (Porvair cat. no. 219004) and shaken at 12 °C for 30 min at 800 rpm in a thermomixer (Eppendorf), placed at −20 °C for 30 min, and thereafter spun at maximum speed in an Eppendorf 5804R centrifuge with an A-2-DWP swing-out rotor (2250*g*) for 60 min at 4 °C. 600 μL portion of the supernatant was transferred to a new deepwell plate with the Bravo liquid handler. After transfer was finished, the receiver plate was dried in a Labconco Centrivap (Labconco Corp.) lyophilizer at 20 °C overnight. The resulting pellets were first washed with 50 μL of deuterated methanol, shaken at 800 rpm, 12 °C for 5 min with the thermomixer, dried in the lyophilizer again for 1 h, and then dissolved in 200 μL of buffer A (37.5 mM sodium phosphate, pD 6.95, 100% D_2_O, 0.02% NaN_3_, 500 μM DSS-*d*_6_, 1 mM imidazole) per well by shaking at 12 °C, 45 min, 800 rpm in the thermomixer. Samples were spun down briefly before 180 μL of each sample was transferred to 3 mm NMR tubes with a SamplePro L liquid handler (Bruker BioSpin). Samples were kept at 4–6 °C during preprocessing and until data acquisition. For an overview of the sample preparation workflow, see Supplemental Figure 1.

### Additional K_2_-EDTA Plasma Tube Tests

A mixture of alanine, butyrate, propionate, and acetate, each at 50 μM concentration in buffer A, with a total volume of 2 mL, was added to four EDTA tubes, inverted twice, and incubated similarly as described above for the plasma samples, i.e., 1 h at 4 or 22 °C or 30 min each at 4 or 22 °C in succession, both possibilities. A tube with only buffer A was also incubated at 22 °C for 1 h. After incubation, 600 μL of each incubated sample and a negative control of buffer A which had not been in contact with an EDTA tube was transferred to 5 mm SampleJet NMR tubes and sealed with POM balls (Bruker BioSpin).

### NMR Data Acquisition

For methanol-precipitated plasma samples, 1D ^1^H NMR data was acquired using an Oxford 800 MHz magnet equipped with a Bruker Avance III HD console and a 3 mm HCN cryoprobe. Sample racks were handled and kept at 6 °C in a SampleJet sample changer before being put into the spectrometer. For 1D ^1^H data, the pulse sequence “zgespe” was used, entailing water suppression through excitation sculpting including a perfect echo element. The acquisition time was 2.04 s, and the relaxation delay was 3 s. Data was acquired in 128 scans and 64k data points. The spectral width was 20 ppm, and the acquisition temperature was 298 K. For the K_2_-EDTA tube tests, a Bruker 600 MHz Avance III spectrometer equipped with a 5 mm BBI room temperature probe was used. 1D ^1^H data was acquired with the “noesygppr1d” pulse sequence using 32 scans collected into 64k data points, a spectral width of 20 ppm, an acquisition time of 2.726 s, and a relaxation delay of 4 s for a total experimental time of 3 min 4 s. Acquired data was processed in TopSpin 3.5pl6 (Bruker BioSpin), including exponential line broadening of 0.3 Hz prior to Fourier transform, automatic phasing, baseline correction, and referencing to the DSS-*d*_6_ signal.

### Analysis of Spectral Data

One participant completely lacked samples with short preanalytical handling time and was therefore excluded, leaving 14 participants contributing with one sample and 13 participants contributing with samples from two occasions, giving in total 929 samples. Data was imported into MatLab 2020b (MathWorks, Inc., Wilmington), aligned with icoshift 1.2^22^ and 325 peaks were visually localized and integrated down to an approximated baseline using in-house developed MatLab routines. The resulting integrated data was normalized with probabilistic quotient normalization^23^ and peaks were annotated with the use of ChenomX 8.3 (ChenomX, Inc., Edmonton, Canada) and the human metabolome database (www.hmdb.ca).^24^ Principal component analysis (PCA) and orthogonal partial least-squares-discriminant analysis (OPLS-DA) models were calculated within SIMCA (17.0.0.24543, Sartorius Stedim) and analyzed in SIMCA and MatLab.

**Figure 1:**
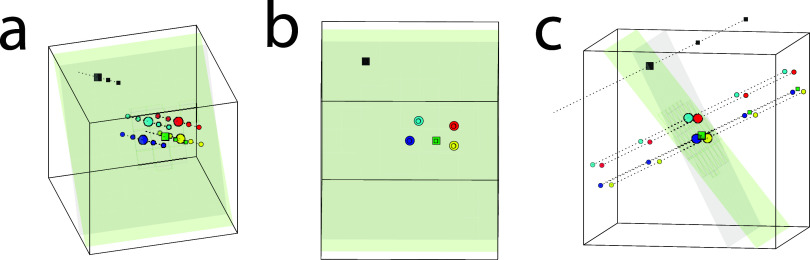
With PCA, a sample metabolite concentration matrix can be reorganized into its principal components, and be back-calculated if the number of components are enough and *R*^2^*X* is 100%. The maximal number of components is determined from either the number of samples or metabolites, whichever is the lowest. (a) Three-dimensional PCA score plot illustrating all components. Four participants (blue, red, yellow, cyan) with three samples each, drawn at the same participant visit, one having short preanalytical handling time and unaffected metabolite concentrations (large circles), and two with longer preanalytical handling times with affected metabolite concentrations (small circles on the right side of the unaffected sample) which have followed their respective preanalytical handling paths (dotted lines) until measurements. The data matrix also includes one calculated “mirror sample” for each sample having longer preanalytical handling time with opposite concentration differences with respect to the unaffected metabolite concentrations (small circles on the left side of the unaffected sample). These make sure that each participant’s mean group value corresponds to the sample with short preanalytical handling and is in a region with allowed concentration profiles not influenced by preanalytical handling (gray area). Two participants’ corresponding samples are predicted onto the model (squares). (b) Viewed at some angle of score space the samples of each participant gather closer together since the preanalytical handling trajectories are similar. An OPLS-DA model using each participant as its own group approximates the gray area as its component space (green area). (c) The orthogonal components extend into the white preanalytical handling-affected volume. The approximation is better closer to the modeled samples and worse further away, making the accuracy of the predictions of the green samples better than the gray which risk having a bias.

OPLS-DA models discriminating on participant visits were built using the maximum number of components possible, in this case, the number of participant visits minus one, and enough orthogonal components to describe the systematic variability in the data other than participant visits, i.e., the preanalytical handling variability. Apart from noise, the predictive components then describe the same space as a maximum number of components PCA model of the mean of each participant visit group would. “Mirror samples” were used setting this mean to something that can be considered unaffected by preanalytical handling (Figure 1, Supporting Information). Back-calculated estimates of metabolite intensity values unaffected by preanalytical handling were calculated from the OPLS-DA predictive components. In this respect, using the *Q*^2^ value describing how well the different participant visits are defined compared to each other depending on the number of components is irrelevant, just as a *Q*^2^ value in a PCA of one unaffected sample per participant visit would be. *R*^2^ is used to check that the component space and orthogonal component space are large enough with little data not explained. Within-model precision estimates measuring the consistency between different preanalytical time, temperature, and light conditions for participant sampling occasions when moving preanalytical handling issues to the orthogonal components were made directly in the scores and orthogonal scores, and on back-calculated metabolite concentrations from either the components or original values with the orthogonal components subtracted. Naturally, whether predictions are accurate or not cannot be based directly on score values if predicted sampling occasion group belongings are not included in the model. Instead the calculations were repeated on data from separate participants not included but predicted in the model to capture accuracy and precision in combination in loadings and back-calculated metabolite intensities data. See Figure 1 and the text in the Supporting Information for an illustration and further overview of the analysis approach taken.

A small-scale qualitative analysis of temperature and time effects on short-chain fatty acids (SCFAs) was evaluated by measuring a small number of metabolite intensities with a consequent qualitative assessment.

Precise analysis of light exposure effects to spot any small differences was done using OPLS-DA on matched “light–dark” vs “dark–light” samples and Wilcoxon Signed rank test.

## Results and Discussion

### Sample Preparation

In contrast to our previous study using ^1^H NMR on preanalytical effects on blood sampling,^4^ we chose to not employ the common standard operating procedure (SOP) of diluting plasma or serum samples but rather a methanol precipitation SOP adapted from Nagana Gowda et al.^21^ to fit a workflow using deepwell plates to process large numbers of samples in parallel. The chosen SOP allows detection and quantitation of signals, e.g., short-chain fatty acids (SCFAs), otherwise masked by broad lipoprotein peaks when a dilution protocol is used.

### EDTA Tube Contribution

The chosen K_2_-EDTA tubes contribute propylene glycol, a molecule that is not present naturally in plasma and which, with this knowledge, can be excluded from any study using the same tubes. However, the tubes also contribute formate, acetate, and sarcosine, as well as signals from a number of unknown molecules (Supplemental Figure 2). In accordance with previous studies,^25,26^ this points to that EDTA plasma tubes, especially those with a gel separator, are not ideal for ^1^H NMR metabolomics. Serum or heparin plasma tubes are better choices for avoiding confounding signals.

### Sample Preanalytical Handling Model

We realized from our data that *explicitly* setting plausible variables (e.g., time, temperature, light status) causing metabolite concentration changes in an OPLS(-DA) model can lead to biases, misinterpretations, and inadvertently missing relevant factors already within our well-controlled study. The metabolite concentration changes are not necessarily easily described as a function of time and temperature only but depend on e.g., other metabolite concentrations as well. In addition, in a real-world situation, the temperature and light conditions could change if the sample is moved from one room to another prior to centrifugation, making it less useful trying to predict e.g., a single temperature value with high precision. Instead, we used OPLS-DA models with samples from each participant and sampling occasion being its own group, rather than predefining preanalytical time, temperature, and light condition in the model building. This gathers all samples of each participant almost at the same place in score space letting the difference between them describing their different preanalytical histories be pushed to the orthogonal components or outside the model. To force the orthogonal components to describe immediate centrifugation at the origin, and not only a common path but also the same absolute preanalytical history of all of the participants, we used “mirror samples” making sure that the mean of each participant’s short preanalytical time samples can be considered nonaffected by preanalytical handling (Figure 1). We decided that all samples with preanalytical sampling times less than 15 min, which in practice in almost all cases meant less than 5 min, could be considered good and representative of the original sample space. For each participant, the mean intensity of each variable of these samples was considered as the best approximation of data unaffected by preanalytical handling and used as a mirror point (see the Supporting Information). In this way, each participant got one “mirrored sample” with reverse preanalytical history per original sample, and the model comprised in total 929 + 929 samples. We think a more traditional linear model rather than a nonlinear (e.g., AI) is reasonable since the work presented, and likely also future work, must restrict itself in the number of samples and therefore also in precision. In addition, OPLS-DA has the advantage of an explicit, easy-to-interpret output in terms of scores and loadings. Describing the changes related to preanalytical handling of a sample as following a trajectory in a multidimensional space of metabolite concentrations orthogonal to a space of original sample concentration combinations is attractive due to its simplicity, making OPLS-DA a good choice. The models were built on univariately scaled and centered data since we think it makes sense that errors relate to between-sample variability rather than absolute numbers.

**Figure 2:**
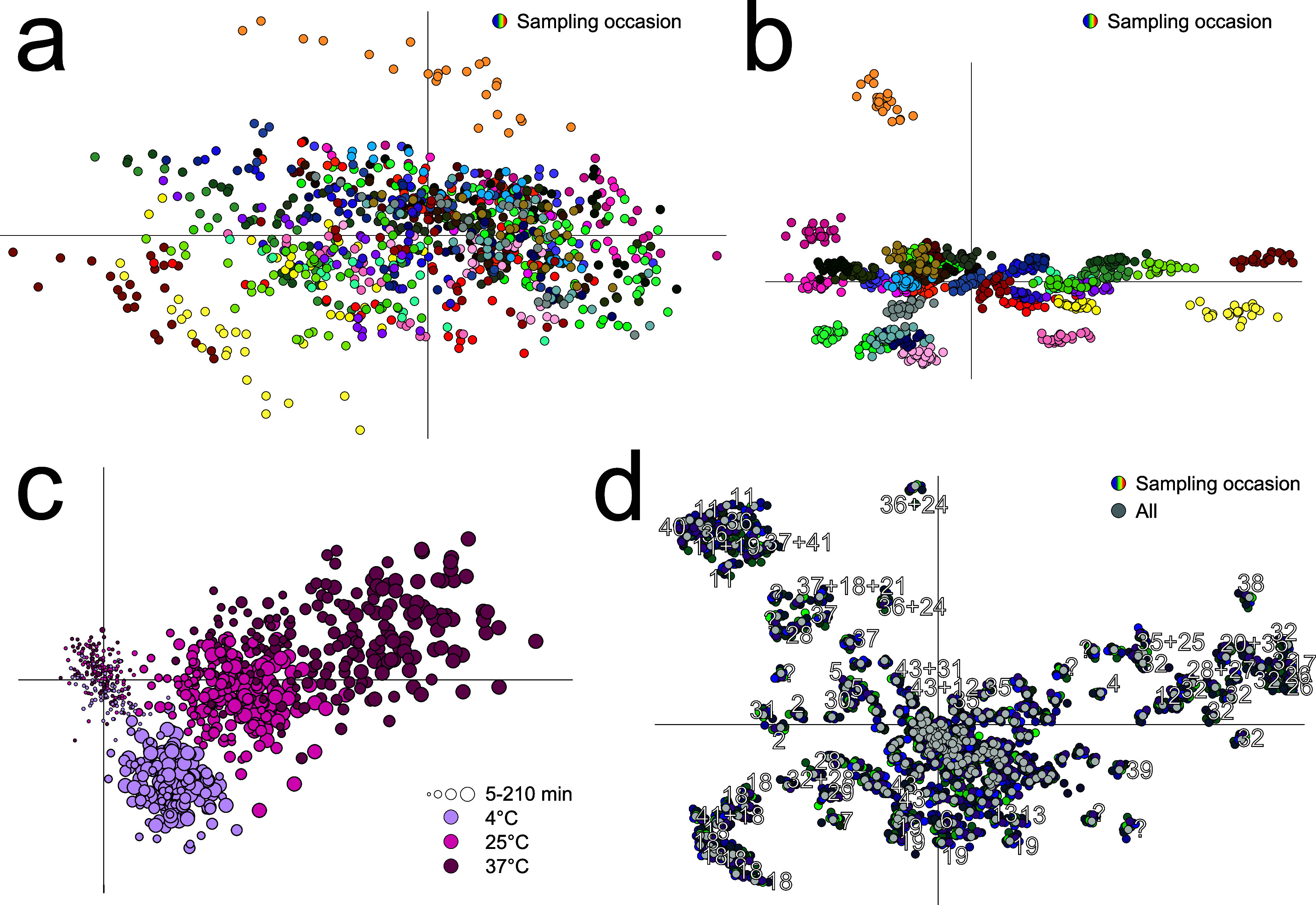
A 39-component, 6 orthogonal component OPLS-DA model, discriminating according to the 40 sampling occasions of 27 participants, of 325 peak intensities from 929 plasma spectra exposed to different preanalytical incubation times at different temperatures either in the dark or in ambient light. Cumulative *R*^2^*X* = 0.878, of which Orthogonal cumulative *R*^2^*X* = 0.235. (a) The first two component scores in a PCA, without the “mirror samples”, illustrating the uncorrected changes occurring during incubation. Samples from a given individual sampling occasion are relatively well localized, but the preanalytical incubation time and temperature trends induce scattering. (b) The first two component scores of the corresponding OPLS-DA model when also the “mirror sample” data is used. For simplicity, the mirror samples are excluded from the plot. The score space is a lower-dimensional projection of the original data matrix and as such, to a large degree filtered from inconsistencies in the induced changes. The positions of the samples are close to where they would have been if they had not been exposed to the induced changes from incubation. (c) The first two orthogonal component scores, *R*^2^*X* = 0.109 and *R*^2^*X* = 0.0618 with “mirrored samples” excluded from the plot. The mirrored sample strategy ensures that samples with short incubation times display low orthogonal score values while longer incubation times give larger orthogonal scores. The patterns of changes are similar for all individual sampling occasions. (d) The corresponding loadings (gray dots) show the metabolite patterns for induced changes. Fourteen separate models for prediction were made for the 14 participants who only contributed samples from one occasion by excluding one individual at a time. Cumulative *R*^2^*X* ranges from 0.873 to 0.878, of which orthogonal cumulative *R*^2^*X* ranges from 0.233 to 0.242. The first two orthogonal components of these models remained similar (colored dots). Numbering refers to identified metabolites listed in Supplemental Table 1.

**Figure 3:**
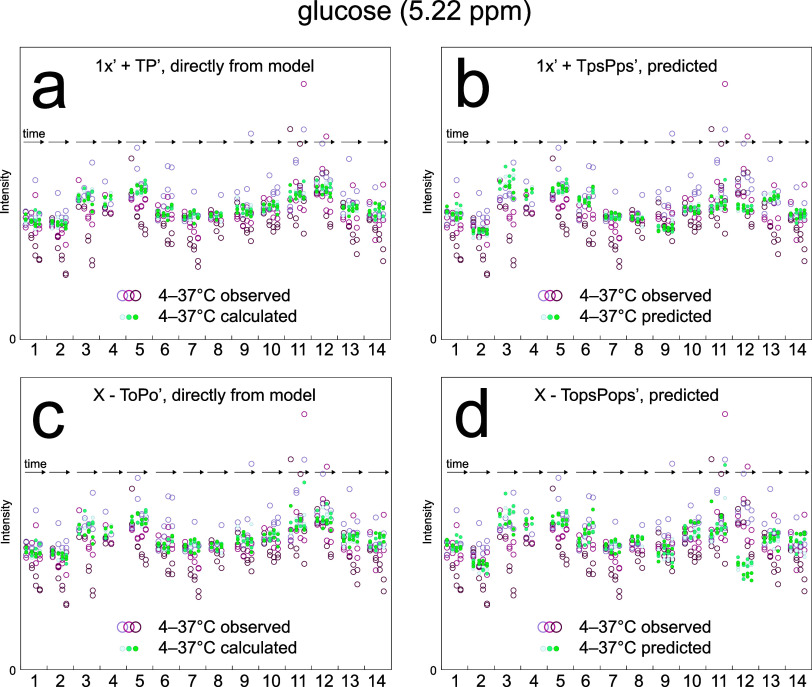
Original metabolite concentrations of the 14 participants with samples only from one occasion were calculated (dots) directly from (a) the components and (c) orthogonal components of the original model, as well as predicted from (b) the components and (d) orthogonal components of the corresponding model where the participant was excluded. Here, glucose is shown (see Supplemental Figures 4–17 for other metabolites). The “0” on the relative concentration metabolite axis shows zero concentration. The calculated values are consistent for each participant and also agree well with the observed values (circles) for nonincubated samples (far left of each participant), independent of using scores or orthogonal scores. The predicted values are also consistent for each participant but often contain a bias for those participants with large distances to model and Hotelling’s *T*^2^, e.g., participant 12, making predictions only suitable for those close to the model plane and not far from others in the plane.

An extensively overfitted model using 39 components to maximize the size of a multivariate room spanning the 40 original samples was built to describe an original sample space with all available preanalytical samples (Figure 2 and Supplemental Figure 3). The large number of components maximizes *R*^2^ and minimizes any remaining information outside the model and maximizes the possible number of independent variables reducing the risk of nontrue metabolite concentration correlations between these. Counterintuitively the *Q*^2^ value describing how well the different participants’ samples are separated from each other is not relevant. The raw data is not overfitted, just rotated into a particular direction and viewed in a lower-dimensional space, where correlations of the variables have been introduced since the number of components is lower than the number of variables. This makes sure that not all metabolite concentration combinations are allowed in the original sample space. The accuracy and precision can best be estimated from predictions using the model. Already within the model, it is possible to get an approximate idea about its precision. Independent of their preanalytical handling, each participant’s samples should be positioned almost at the same place in score space, pushing almost all preanalytical handling-related changes into the orthogonal space similar to all participants. Also, back-calculated metabolite concentrations should be consistent for each participant’s samples and similar to those with a short time to centrifugation. Indeed, adding orthogonal scores shows consistent preanalytical handling patterns between participants. This means that it is possible to estimate the preanalytical handling of a particular observation from nearby observations in the orthogonal space with known preanalytical history. The first two orthogonal components capture most of the temperature and time effects, while adding a third captures methanol precipitation dilution variability and data normalization. Adding more components had a limited impact. The fourth does not seem to have an obvious preanalytical explanation. The fifth and sixth orthogonal components show a preanalytical handling effect at especially 37 °C unique for some, especially one, participant due to a large increase in one unknown metabolite with peaks at 1.36/28.75 ^1^H/^13^C ppm and 3.30 ^1^H ppm. The ambient light conditions during sampling did not have any obvious effect on the model. Each participant’s samples gather in the scores of the model, the ones being more in the center seemingly randomly depending on preanalytical handling while some participants with more deviating metabolite patterns still have small but obvious correlations with preanalytical handling (not shown). Back-calculating the individual molecule concentrations either from the model or from subtracting the original data with the orthogonal model, i.e., in practice exclude or include the distance to model (DmodX), shows that six orthogonal components largely suppress preanalytical handling effects in the data (Figure 3a,c and Supplemental Figures 4–17a,c). Prior to modeling, most peaks show small preanalytical handling differences in intensity, although it could be expected that many metabolite concentrations are invariant during the up to 4 h preanalytical handling period. Probably this can be attributed to the precision in the intensity calculations in combination with the normalization of the data after methanol precipitation with most normalization constants between 0.9 and 1.1 but some deviating up to 30% from average, making otherwise quantitative NMR measurements less so. This spills over into the model, with many metabolites having small deviations from zero in the orthogonal loadings risking minor accuracy problems of the corresponding back-calculated metabolite concentrations. A more robust protocol, e.g., as would be possible from using the Bruker IVDr SOP, involving a stricter quality control, would probably be beneficial also for concentration estimations after modeling. In fact, even three orthogonal components are sufficient to obtain relatively good precision in the preanalytical handling effect estimations (not shown). Also in a larger study, a smaller number of orthogonal components should be sufficient to describe the major preanalytical handling effects as long as it is not enlarged by other surprising effects like the 1.36/3.30 ppm molecule mentioned above or samples with very different metabolite concentration change patterns, e.g., due to disease.

**Figure 4:**
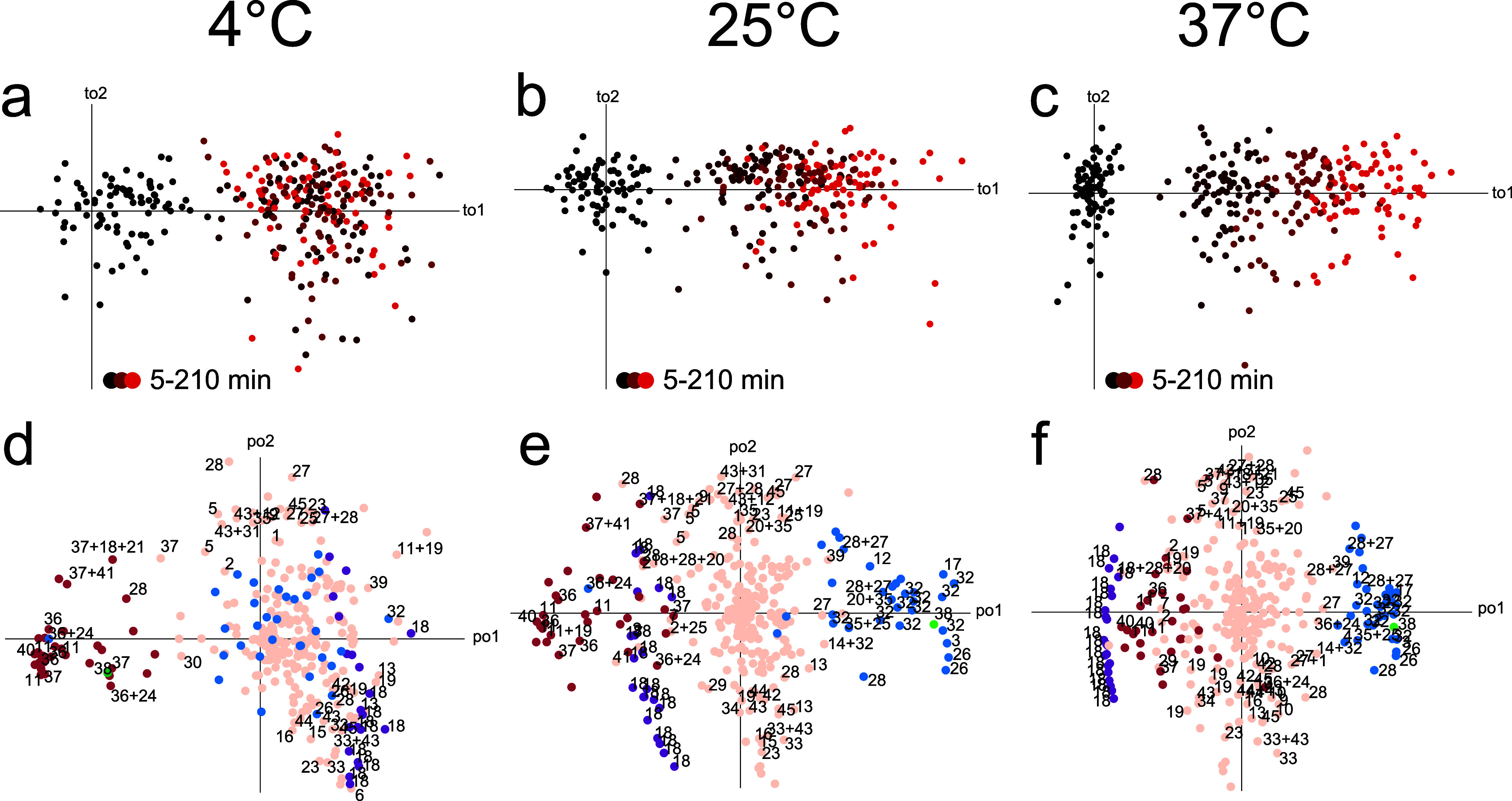
Scores (a–c) and loadings (d–e) of OPLS-DA 39-component 2 orthogonal component models of 4 °C, 25 and 37 °C data, cumulative *R*^2^*X* = 0.861, *R*^2^ = 0.864, *R*^2^ = 0.876, show four distinct groups of characteristic changes depending on incubation temperature; short-chain fatty acids (red), the glycolytic intermediate pyruvate (green), glucose (purple), and a group of molecules including lactate and ornithine (blue). Metabolite numbering is according to Supplemental Table 1.

The main model, as well as separate models of each temperature, show four distinct temperature-dependent preanalytical handling characteristics for some SCFAs, the glycolytic intermediate pyruvate, glucose, and a group of molecules including lactate and ornithine (Figure 4). The glucose, lactate, pyruvate, and ornithine changes are consistent with earlier studies.^4,6,27^ At 4 °C essentially nothing happens except for an initial quite dramatic decrease in SCFAs and pyruvate (Figure 4a,d). The orthogonal scores of the 5 min samples are separated from the others. At 25 °C, the SCFAs also decrease but to a lesser extent (Figure 4b,e). Pyruvate, lactate, and ornithine increase, and glucose decreases. The 5 min, 1.5 h, and 3.5 h samples are separated in orthogonal space with the 2.5 h overlapping both the 1.5 and 3.5 h samples giving an idea about how fast the metabolite concentration changes are and the resolution of the model, i.e., how precisely can the preanalytical time before centrifugation be estimated. At 37 °C glucose decreases the most, SCFAs only slightly (Figure 4c,f) and pyruvate, lactate, and ornithine increase more rapidly than at 25 °C. The 2.5 h samples are at this temperature separated from both the 1.5 and 3.5 h samples.

### Prediction Modeling

**Figure 5:**
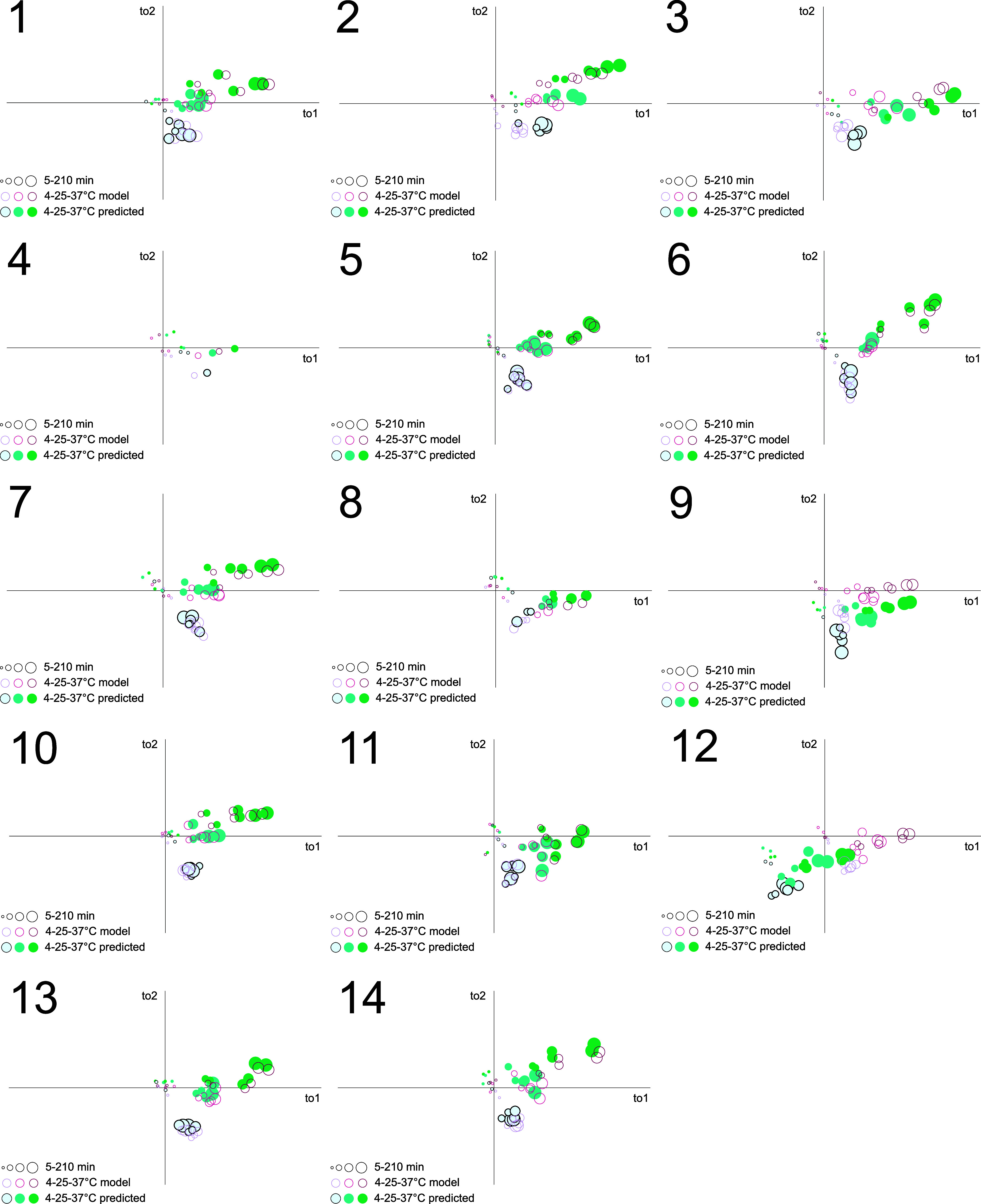
First two orthogonal scores of separate prediction models for the 14 participants who contributed with samples from one occasion only, with component *R*^2^*X* values ranging from 0.108 to 0.112, and from 0.0607 to 0.0632. The scale of the axes is the same in all 14 figures, and the positions of the time and temperature regions are directly comparable. The relative positions between the orthogonal scores for each participant whether the samples are included in the OPLS-DA model or excluded and predicted are almost conserved, meaning that the orthogonal score “preanalytical handling space direction” is well described. However, when the predicted data of an individual does not fit well into the model in terms of distance to model and in particular Hotelling’s *T*^2^ range, e.g., participant 12, the orthogonal scores are often biased and the predicted preanalytical handling temperature and time are clearly outside also the between participant precision of the model.

We predicted all samples’ metabolite concentration changes and applied adequate corrections for each of the 14 participants who had samples only from one occasion, using separate OPLS-DA models of the remaining 39 original samples and 38 regular and 6 orthogonal components where the predicted participants’ data were excluded. The orthogonal components successfully subtract the differences related to preanalytical handling between each participant’s samples. The “preanalytical handling direction,” i.e., “the derivative” of the orthogonal space, is almost intact and each participant’s predicted sample positions closely localized in the model plane. The precision of the metabolite concentration predictions is also good. However, leaving a participant’s samples outside a model to predict them instead sometimes results in consistent biases in both the components and the orthogonal components (Figure 5 and Supplemental Figures 18, 19).

The prediction errors also translate into errors in the predicted metabolite concentrations (Figure 3b,d and Supplemental Figures 4–17b,d) motivating a more careful examination into causality to find possibilities for improvement. The 325 variables used are larger than the underlying number of measured metabolites and therefore not determine the dimensions of the system. But the metabolites are clearly more than the 39 groups spanning the OPLS-DA models, meaning that the OPLS-DA models assume linear dependencies between the metabolites such that new predicted samples can be outside the model plane with a remainder not correctly predicted. Preferably the number of model dimensions should reflect the number of metabolites which is approximately twice compared to now but that would require more participant visits. Most importantly, additional participant visits would make the data set less sparse and with a wider distribution. There is now an obvious correlation between Hotelling’s *T*^2^ versus the concentration accuracy in predictions (Supplemental Figure 20). Good predictions are obtained when there are samples that are relatively similar in the model. Since the idea is to replace a set of measured concentrations of an individual’s sampling occasion, which could be thought of as a position in a multivariate space, with another coordinate in this space but within the original sample region, both the preanalytical handling trajectory to follow into the allowed space and the shape of the space itself needs to be relatively correct. The trajectory, the orthogonal components, seem to be well predicted using data from only a few individuals, and future models mostly need data without preanalytical handling bias to define the score space of the allowed region with higher precision. This more detailed analysis would require a relatively large sample set and is probably the main reason why such data is lacking today although many studies have investigated the preanalytical history effect on various analytical readouts. Preferably these data should be drawn from a wide distribution of people also including unusual sample types such as less controlled diabetics.

The risk of metabolite concentration accuracy errors should be weighed against the possible gains from the prediction. Already our rather crude model, with relatively few underlying samples at hand, shows that glucose, lactate, fucose, ornithine, and taurine gain from prediction, when the predicted time to centrifugation is 2.5 h or more at 37 °C but also at 25 °C. This holds as long as the distance to model and Hotelling’s *T*^2^ does not differ a lot compared to the samples in the model. Other molecules such as 2-oxoisocaproate, acetate, choline, glutamine, mannose, propylene glycol, and pyruvate would also be beneficial to predict using a model with more samples as outlined above. Already from the present model, however, qualitative estimates of these measured concentrations can be given as “probably too high” or “too low.”

### Analysis of Temperature and Time Effects on SCFAs

**Figure 6:**
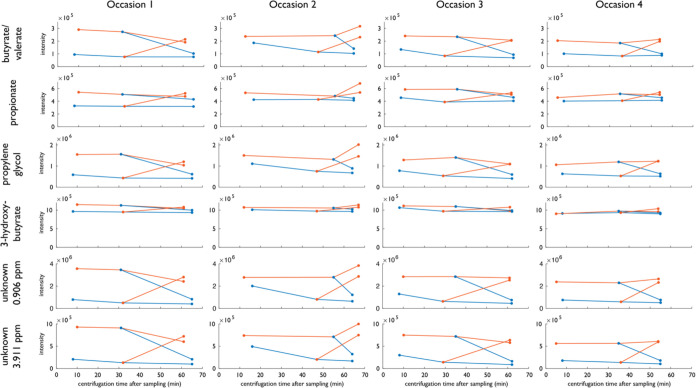
Effect of incubation at a cold temperature before centrifugation. Samples from the same participant sampled at four different occasions were placed at 4 °C (blue) or 22 °C (red) for approximately 30 min, 60 min, or a sequence of 30 + 30 min at 4 °C followed by 22 °C, or vice versa. Butyrate/valerate, propionate, propylene glycol, 3-hydroxybutyrate, and an unknown molecule decrease at 4 °C but at least partly recover at 22 °C.

The decrease in some SCFAs, especially propionate and butyrate, prompted additional investigation (Figure 6). The decrease was most pronounced at 4 °C. The extra experiments with alternating temperature showed that the decrease observed in SCFAs at 4 °C is reversible; i.e., metabolite intensity is partly recovered by increasing the temperature to 22 °C prior to centrifugation. The decrease is rapid since the differences are seen already at the first measurement point after ∼10 min. The decrease in SCFAs at cold temperatures is not seen in the control experiment with a mix of SCFAs alone without plasma in the same type of EDTA plasma collection tube (Supplemental Figure 2a). The reversibility of the changes seen in SCFAs suggests that it is a real biological effect connected to interaction with plasma macromolecules, e.g., proteins, and/or cellular membranes. If this is a general phenomenon as a response to cold temperature, it might be an indication that certain SCFAs are used as natural cryoprotectants, a finding that needs further investigation and is outside the scope of this work. The concentration variability of SCFAs depending on the temperature in a sample is from the analysis perspective worrying. It is obvious that these concentrations are often underestimated since the temperature just prior to sample processing often is lower than body temperature if tubes have been stored in a fridge or even at room temperature. If this temperature is not the same for all samples in a study it could easily introduce a bias.

### Analysis of Light Exposure Effects

**Figure 7:**
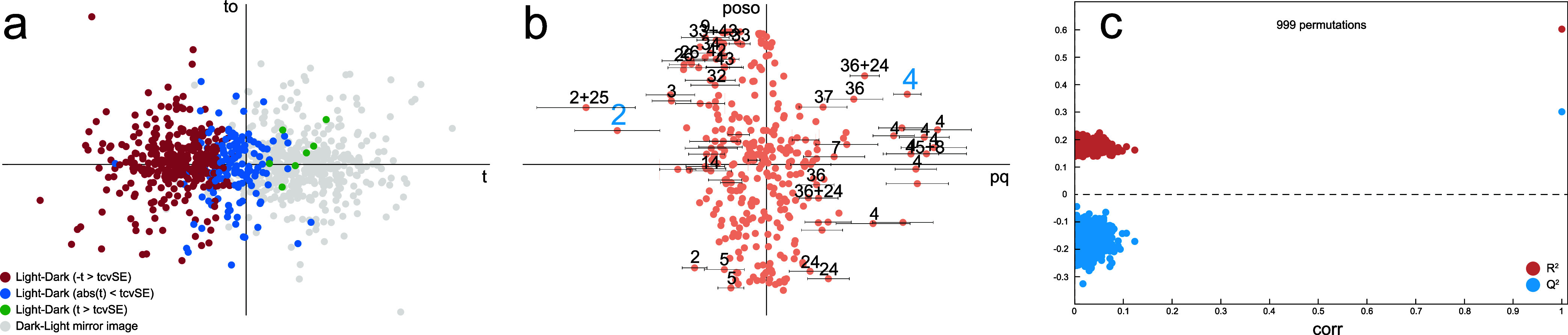
**(a**) Score plot of an OPLS-DA model with three orthogonal components of light–dark (red, blue, or green, depending on size compared to the cross-validated standard error) vs dark–light (gray) of all 470 available light and dark sample pairs shows a minor separation of the groups. Cumulative *R*^2^*Y* = 0.602 and *Q*^2^ = 0.301. (b) The corresponding loading plot showing 2-oxoisocaproate (2) increase and 3-hydroxybutyrate (4) decrease in ambient light compared to the dark. The particular peaks used in univariate analysis are enlarged and colored blue. The error bars show the cross-validated standard error. (c) Cross-validation was performed using seven groups, always including samples from the same participant visit in the same group. A permutation test shows a much higher cumulative *Q*^2^ value for the model than that in any of 999 permutations.

It is well-known that bilirubin in plasma can be affected by light exposure but only after extended storage at ambient or above ambient temperature.^28^ To the best of our knowledge, no report has been published on the effect on plasma metabolites due to storage in ambient light. It is true that light exposure in the present work does not seem to have any effect in the preanalytical handling estimation and prediction models, but has a very minor effect when specifically comparing the available 470 matched light–dark vs dark–light sample pairs in OPLS-DA models (Figure 7). Permutation tests and models on different subsets leaving the remainder as independent data to test on show that these differences are real but minute. The result is consistent with various model subgroups of people, temperatures, and times to centrifugation (not shown). For most of the participant visits, 3-hydroxybutyrate and 2-oxoisocaproate are relatively lower and higher in concentration, respectively, when exposed to light in the majority of measured light vs dark pairs. A two-sided Wilcoxon signed rank test of 3-hydroxybutyrate (1.19 ppm) and 2-oxoisocaproate (2.60 ppm) show uncorrected *p*-values of 2.0 × 10^–6^ and 1.0 × 10^–3^, respectively, meaning that 3-hydroxybutyrate clearly correlates with light exposure but the 2-oxoisocaproate finding might be incidental.

### Other Considerations

To avoid misinterpreting any measurement batch effects for preanalytical sample handling changes, all samples, with few exceptions, were randomized for each participant and sampling occasion and measured immediately after each other. Consequently, the data set does not allow any precise batch effect control, and instead batch variation will go mainly into the individuals’ sampling occasion modeled scores. We do not think there is any reason to believe that we have any major batch effects, but it should be noted that we for a very small number of peaks saw a change in peak intensity depending on time in the cooled SampleJet sample changer prior to NMR measurement.

### Limitations of the Present Study

The main limitation of this work is that the models are built on sample data from a biased and small number of participants. To go beyond this pilot stage and make a general model suitable for the assessment of existing plasma samples in biobanks, a much larger sample set is needed. Such a sample set should cover not only a bigger “participant space” of samples with short preanalytical handling time but also include disease states, different preanalytical handling times under normal lab conditions, variation in storage conditions, i.e., in particular freeze–thaw cycles, different sampling tubes, *etc*. The choice of a methanol precipitation SOP also limits the facile extension of such a sample set to those laboratories where a similar lab automation setup is available. Repeating the present study with, e.g., the strict Bruker IVDr protocol would be beneficial to extend it but would also mean losing out on SCFA data as those signals are totally covered under broad lipoprotein/lipid signals in diluted plasma.

## Conclusions

The preanalytical handling metabolite concentration change patterns, “the derivative” of sample concentrations, are largely conserved between samples. Biologically allowed ratios between certain metabolites when a sample is drawn, the relative metabolite concentrations at “time zero,” are also limited. This makes prediction of what temperature and time exposure a plasma sample has been exposed to before centrifugation possible. We show that it is possible to predict metabolite concentrations of a corresponding original sample using an OPLS-DA model, making comparisons between differently handled samples possible and analyses of biobanked samples from different sources more feasible than today. In this context, it is tempting to suggest future models used by the community, built on a large shared data set where relevant subsets can be used when motivated, for example, depending on what sampling tube type was used.

If samples are stored in ambient light or not prior to centrifugation has no practical significance for all metabolites, including also 3-hydroxybutyrate and 2-oxoisocaproate since the observed effect is very minor compared to all other preanalytical influence. Additionally, if SCFAs are of interest in a study, the recommended sampling SOP should be to keep sample tubes at room temperature before centrifugation to ensure correct SCFAs concentrations without unnecessary preanalytical handling. Without knowledge about the actual sampling protocol used for a given sample set requested from a biobank, any resulting quantification of SCFAs (including acetate in the case of EDTA plasma) should be handled with caution, as there might be confounding bias due to sampling conditions. In an ideal case but still practical in terms of everyday work in clinical chemistry laboratories, plasma sampling should be done immediately at RT, otherwise storage at 4 °C followed by warming to RT just before centrifugation is desirable to represent the metabolome reasonably accurately to the state it was at blood draw. The warming of sample tubes before centrifugation goes against current recommendations^14,15,29^ but it is also the first time to our knowledge that the decrease of SCFAs in plasma at low temperatures has been observed.

With the present work and previous studies, the inherent reproducibility and quantitative aspect of ^1^H NMR spectroscopy has demonstrably shown its suitability for facile quality control of serum and plasma preanalytical variability. However, there is no established standard on sample preparation SOP or comprehensive models taking into account all potential variables at play, i.e., matrix, sampling tube, temperature, centrifugal force, *etc*. What we propose in this work is that all preanalytical variability affecting the metabolome can be captured in the orthogonal space without explicitly stating what a given sample has been subjected to. Thus, a future sample can be classified in this “preanalytical handling space” as one that displays a metabolite profile consistent with e.g., 25 °C storage for 2 h or 37 °C for a shorter time, even though the actual conditions were not known. To be adopted into practical use, however, this approach requires the model to be extensively expanded to cover other variables such as the mentioned tube types, serum as well as different types of plasma, and representation of not only healthy subjects but also disease states.

## Supporting Information Available

Background to choice of data modeling approach (Supporting Table 1) (Supporting Figures 1–20) (PDF)
