# Supplementary material for: Preanalytical (Mis)Handling of Plasma Investigated by 1H NMR Metabolomics
Source: ACS Omega. 2024 Nov 27;9(49):48727–37. doi: 10.1021/acsomega.4c08215 (PMC11635485; doi:10.1021/acsomega.4c08215)
Supplement: Supplementary file 1 — ao4c08215_si_001.pdf [file ao4c08215_si_001.pdf]

Pre-analytical (mis)handling of plasma investigated by ^{1}H-NMR metabolomics

Daniel Malmodin[1,5*], Anders Bay Nord[1], Huma Zafar[2], Linda Paulson[2], B. Göran Karlsson[1], Åsa Torinsson Naluai[2,3,4]

[1]Swedish NMR Centre at the University of Gothenburg, Gothenburg, Sweden; [2]Biobank Väst, Gothenburg, Sweden; [3]Biobank Core Facility; [4]Institute of Biomedicine, Sahlgrenska Academy, University of Gothenburg, Gothenburg, Sweden; [5]National Bioinformatics Infrastructure Sweden (NBIS)

* corresponding author: daniel.malmodin@nmr.gu.se

Abstract

The pre-analytical handling of plasma, how it is drawn, processed, and stored, influences its composition. Samples in biobanks often lack this information and consequently important information about their quality. Especially metabolite concentrations are affected by pre-analytical handling making conclusions from metabolomics studies particularly sensitive to misinterpretations. The perturbed metabolite profile, however, also offers an attractive choice for assessing the pre-analytical history from the measured data. Here we show that it is possible using Orthogonal Projections to Latent Structures Discriminative Analysis to divide plasma NMR data into a multivariate 'original sample space' suitable for further less biased metabolomics analysis and an orthogonal 'pre-analytical handling space' describing the changes occurring from pre-analytical mishandling. Apart from confirming established pre-analytical effects on metabolite levels, *e.g.* the consequent changes in glucose, lactate, ornithine and pyruvate, the sample preparation protocol involved methanol precipitation which allowed the observation of reversible changes in short-chain fatty acid concentrations as a function of temperature.

Keywords: sample handling, short-chain fatty acids, OPLS-DA

Supporting information

Table of contents

Background to choice of pre-analytical data modelling approach

Model building and prediction

Supplemental table 1

Supplemental figures 1-20

Background to choice of pre-analytical data modelling approach

OPLS-DA originates from PCA. PCA is a dimensionality reduction technique capturing the main trends in a dataset matrix of observations with associated variables. A dataset matrix can be thought of as a sparsely populated space with number of dimensions equal to the number of variables and the observations positioned according to their variable values. In PCA, after variable unit variant scaling and mean centering, a rotation of the space is calculated spanned by so called principal components. The axes in the rotated space are orthogonal to each other but do not represent individual variables. The fraction of the total variability each principal component explains is equal or lower than the previous and quantified in a number R^2X. Cross validation consistency calculations comparing subpopulations and a value Q^2 show if the principal components are describing general trends or are more random. Usually, the number of observations is smaller than the number of variables. Still, by calculating the number of observations minus one principal component the entire original dataset is preserved and can be back-calculated using the coordinates of the transform, the so-called loadings, and the position of the observations in the principal component space, the so-called scores, together with the scaling and mean centering values used prior the PCA calculation. But more common is to use principal components up until the Q^2 value calculation is starting to drop. How much omitted principal components affect individual observations in terms of unexplained variability is quantified in a distance to the model parameter DmodX. A relative measure describing how far from the average an observation is compared to the others in the different principal components and overall model plane is quantified in the parameter Hotelling's T^2. A low DmodX value together with a low T^2 value mean that the observation is both well defined by the model and not very different from the average.

New observation data can be mapped, 'predicted', onto an existing PCA model. Assuming a larger number of variables than principal components, the new observations are unlikely to exactly fit the model but will, just as when all principal components are not used, have unexplained variability seen in the parameter DmodX. How similar the new observation is to the observations building the model is also seen in the T^2 value. In general, it is only meaningful to map a new observation onto a model if the two values are reasonably low such that the observation is not an outlier.

OPLS-DA is similar to PCA. Just like in PCA the dataset matrix is unit variance scaled and mean centered, observations are mapped from the variable space to another often lower dimensional space spanned by orthogonal vectors. R^2X is calculated showing the fraction of the total variability being explained in the model,

and it is possible to back-calculate the entire original dataset if the vector space is large enough which is rarely the case since the model is usually rounded off as in PCA. The difference is that in OPLS-DA components are calculated to maximize the correlation between the data matrix and another user defined matrix describing the group belongings of the observations, and 'orthogonal components' are calculated to capture variability not correlated with the latter. The Q^2 value now describes how well the user defined groups are separated in the data matrix. Loosely, the idea is to rotate the multivariate PCA space into a component space where observations belonging to the same group are as close as possible and an orthogonal space where other than group belonging correlations shows up. Usually, the component space is analyzed to find significant differences between the user defined groups ideally from mapping, 'predicting', new observation data. The orthogonal space is often less studied.

In the present work OPLS-DA is interpreted differently. Time, temperature and light are not groups regressed onto but instead the different participant visits are. OPLS-DA is only used to make a convenient rotation of a PCA space. The assumption is that measured fresh blood samples without pre-analytical handling effects, here called mirror points, (a) only have metabolite concentrations within some allowed limits, and (b) some measured metabolite concentrations are correlated effectively making the number of independent variables smaller than the measured metabolites. (a) means that a PCA component space of such samples also will have allowed limits within the model scores and (b) means that the number of components necessary to fully describe the whole dataset will be smaller than the number of measured metabolites. An OPLS-DA of only mirror points, each being its own group, and without orthogonal components will simply be a PCA with fewer than the number of measured metabolites dimensions where the positions of the mirror points in score space show allowed blood sample regions not affected by pre-analytical handling. The difference between the number of measured metabolites and the number of components can be used as orthogonal components. By adding data of samples exposed to pre-analytical handling and their in the mirror points mirrored samples (see below) to the respective groups in the OPLS-DA, and this time also calculate a sufficient number of orthogonal components, the original component space will remain (almost) intact. Predicted samples which falls within the allowed region of mirror points in component space, and the orthogonal space in practice describing pre-analytical handling, are thought to be well predicted in terms of *e.g.* time and temperature and their pre-analytical handling-affected metabolite original concentrations possible to estimate. It should be noted that we were very limited in number of participant visits making the number of possible components smaller than otherwise desirable. But most importantly, a larger or much larger number of participant visits with short pre-analytical handling times, possible to use as mirror points, would be very useful to better describe the allowed space. It could be that in such a situation it would be beneficial to make models and predictions on only parts of the data similar to the query sample. Together with these also corresponding samples describing typical pre-analytical handling would be useful also if no information of how it has been handled is known. Obviously, if the dataset is large enough also machine learning or other AI approaches could be of interest.

Model building and prediction
An OPLS-DA model divides a dataset (X) into its mean (1x') and variability where two are systematic, either correlated (TP') or orthogonal (ToPo') to the groups therein, and one is the residual variability (E)

$X = 1x' + TP' + ToPo' + E$

We used this equation by applying it on model data and setting the groups equal to the participant visits so that 1x' + TP' describe the original sample and the induced incubation change is represented by ToPo'. First we calculated the mean of each persons and sample occasion samples with 'Time to centrifugation' less than 15 minutes and decided that these should represent coordinates in a 'non-incubated TP' space', their respective mirror points. Second, we calculated the difference between these mirror points and their corresponding samples. By using both the positive and negative deviation from the mirror points*, i.e.* using each measurement twice, we mirrored the data forcing the samples with 'Time to centrifugation' less than 15 minutes to have no or small ToPo' values.

The original data was still used:

x1_ij = (x_ij - mean(x_i)) + mean(x_i) = x_ij

where i is participant, j sample, and mean(x_i) is 'mean of individual i's samples with less than 15 minutes to centrifugation' or simply its mirror point.

In addition, we used ’mirrored data’:

x2_ij = -(x_ij - mean(x_i)) + mean(x_i) = 2* mean(x_i) - x_ij

Predicted induced incubation change is obtained by assuming similarity between the predicted and the model samples in the component and orthogonal component space. Adjusted metabolite concentrations are obtained from calculating either X -TopsPops' or 1x'+TpsPps', depending on whether the residual variability E is added back or not after prediction.

Supplemental table 1. Identified metabolites and corresponding numbering used throughout all figures

**Metabolite**	**Nr**	**Chemical shift (ppm)**
2-hydroxyisovalerate	1	0.824
2-oxoisocaproate	2	2.600
2-phenylpropionate	3	7.348
3-hydroxybutyrate	4	1.187
3-methyl-2-oxovalerate	5	0.884
3-(trimethylsilyl)-1-propanesulfonic acid-d_6	6	-0.009
acetate	7	1.905
acetoacetate	8	2.269
alanine	9	1.463
asparagine	10	2.841
butyrate	11	1.533
choline	12	3.192
citrate	13	2.516
creatinine	14	2.623
ethylenediaminetetraacetic acid	15	3.636
formate	16	8.444
fucose	17	1.235
glucose	18	5.220
glutamine	19	2.437
glutamate	20	2.317
sn-glycero-3-phosphocholine	21	4.315
glycine	22	3.546
histidine	23	7.070
isobutyrate	24	0.879
isoleucine	25	1.001
lactate	26	1.316
leucine	27	0.951
lysine	28	3.015
mannose	29	5.170
methionine	30	2.623
myo-inositol	31	4.060
ornithine	32	3.044
phenylalanine	33	7.417
O-phosphocholine	34	3.205
proline	35	4.131
propionate	36	1.065
propylene glycol	37	1.131
pyruvate	38	2.361
serine	39	3.955
syringate	40	3.909
taurine	41	3.413
threonine	42	4.242
tryptophan	43	7.533
tyrosine	44	6.888
valine	45	1.031

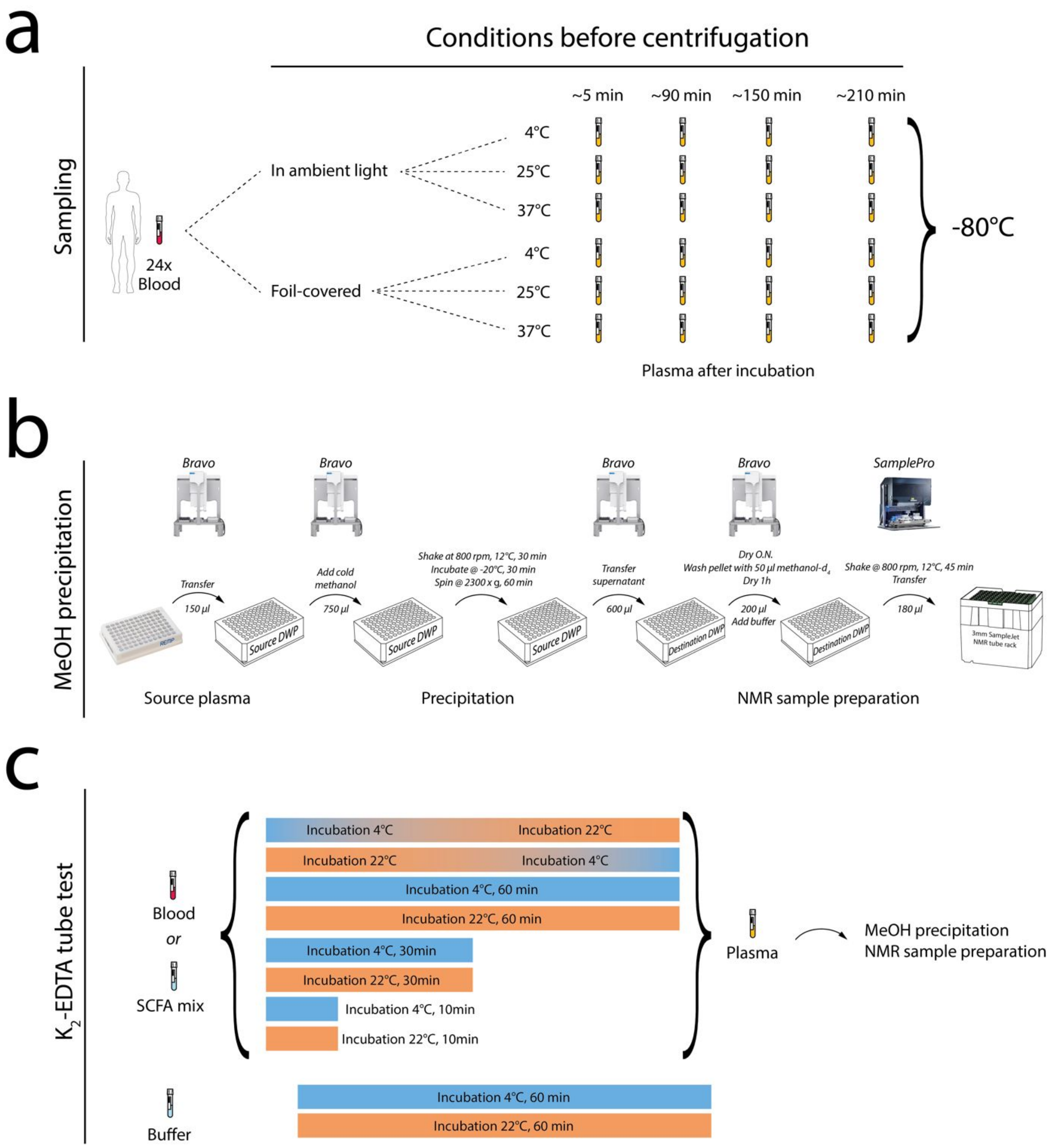

Supplemental figure 1. Overview of the sampling scheme for a given participant at one sampling occasion (a). Flowchart for the methanol precipitation of EDTA plasma samples (b) and the tube tests performed on plasma, a short-chain fatty acid-mix and buffer only (c).

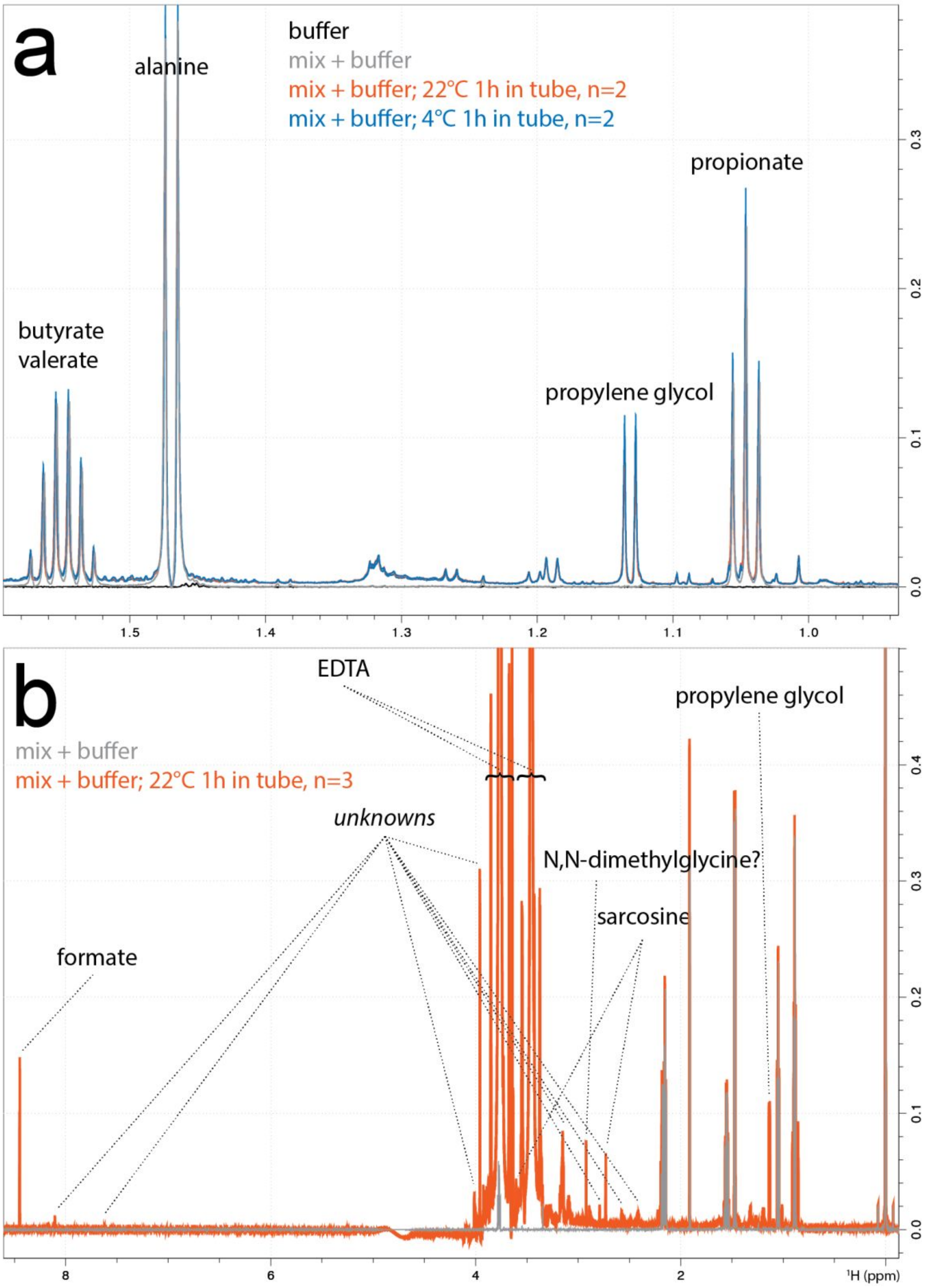

Supplemental figure 2. a) no difference in spectral intensities for SCFAs from an artificial mix when incubated with the K_2EDTA tubes at either cold or ambient temperature **b)** K_2EDTA tubes contribute signals (orange peaks not seen in the reference grey spectrum) to plasma samples.

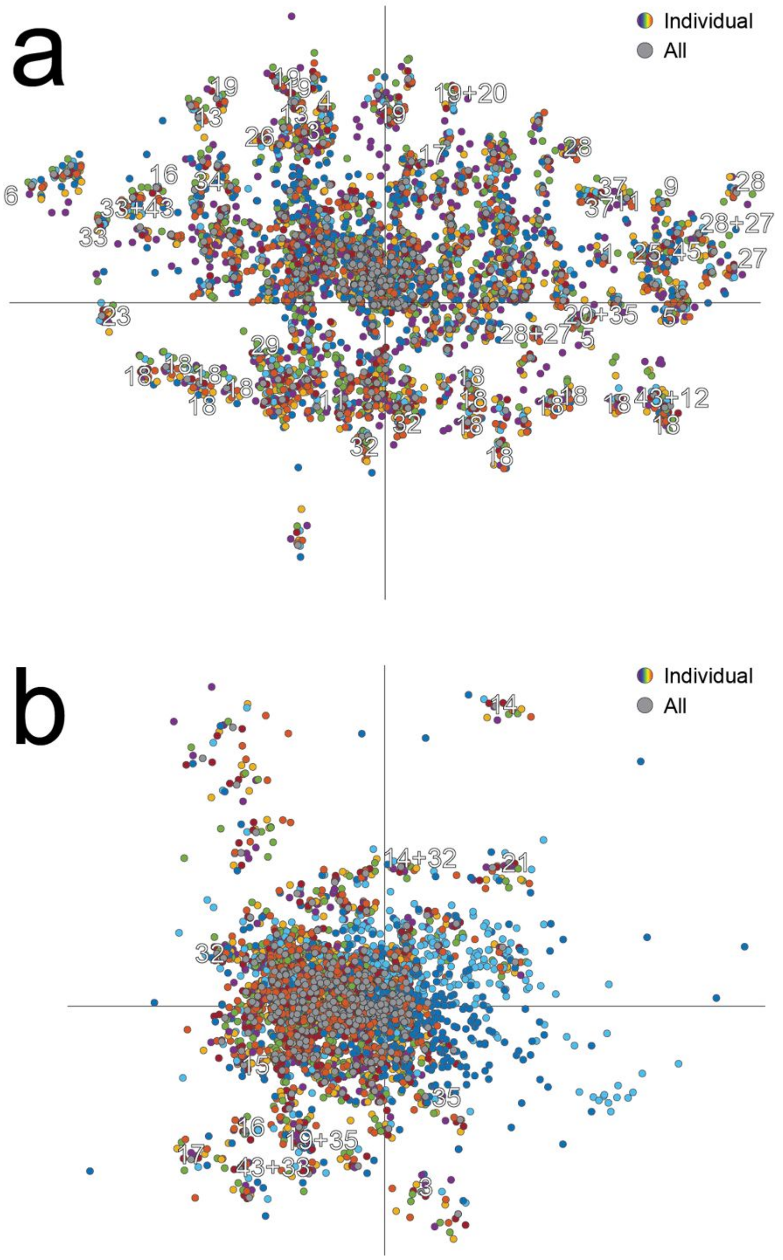

Supplemental figure 3. Same as Figure 2d but orthogonal loadings (a) 3rd vs 4th, and (b) 5th vs 6th. As for orthogonal loadings 1 and 2, 3-6 are also relatively stable independent of model except in two cases where exclusion of a participant makes loadings 5 and 6 differ. This has limited overall impact in the model since the orthogonal score values are small for these components.

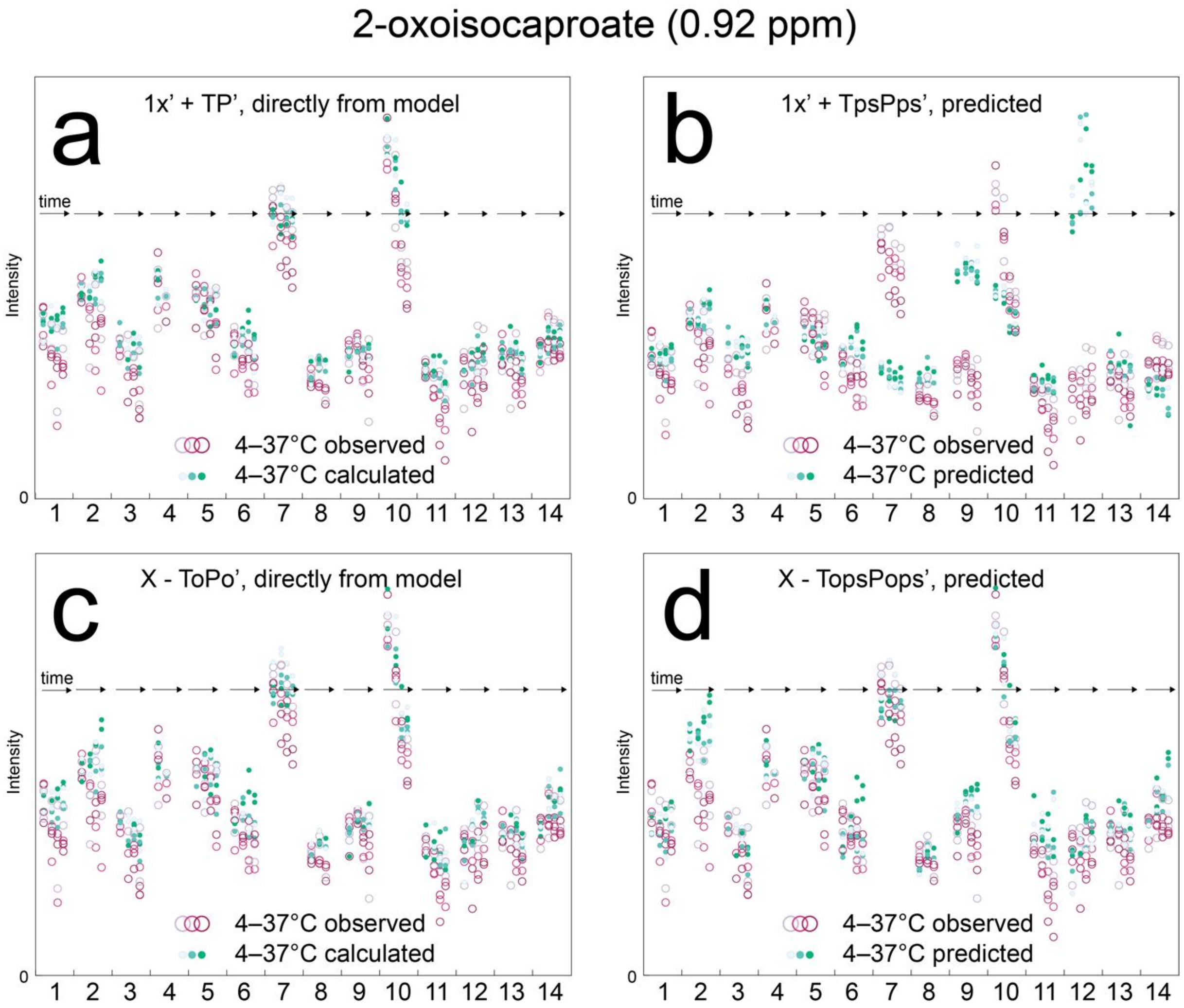

Supplemental figure 4. 2-oxoisocaproate concentrations calculated directly from **a)** the components and **c)** orthogonal components of the original model, as well as predicted from **b)** the components and **d)** orthogonal components of corresponding model where the participant was excluded.

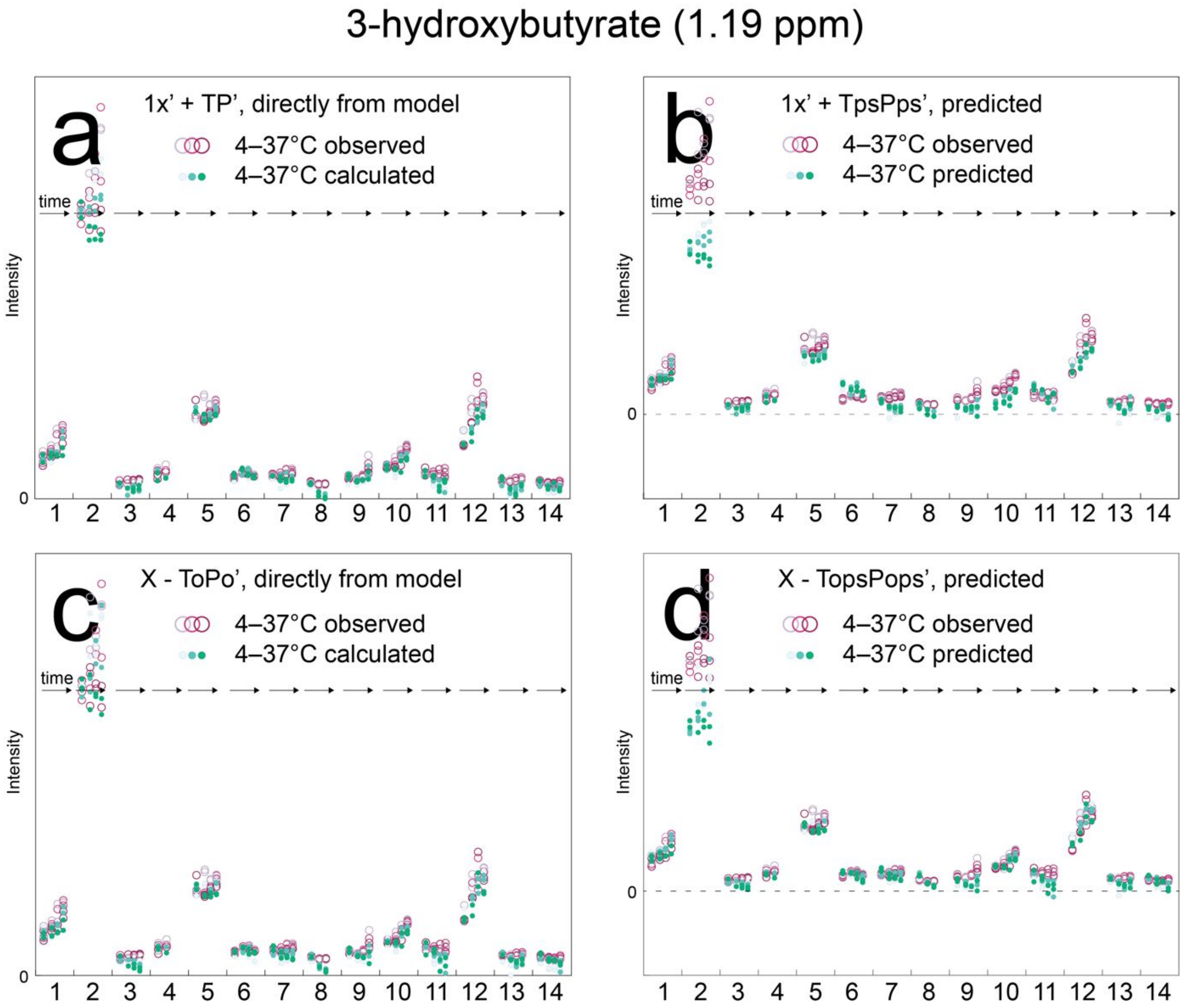

Supplemental figure 5. 3-hydroxybutyrate concentrations calculated directly from **a)** the components and **c)** orthogonal components of the original model, as well as predicted from **b)** the components and **d)** orthogonal components of corresponding model where the participant was excluded.

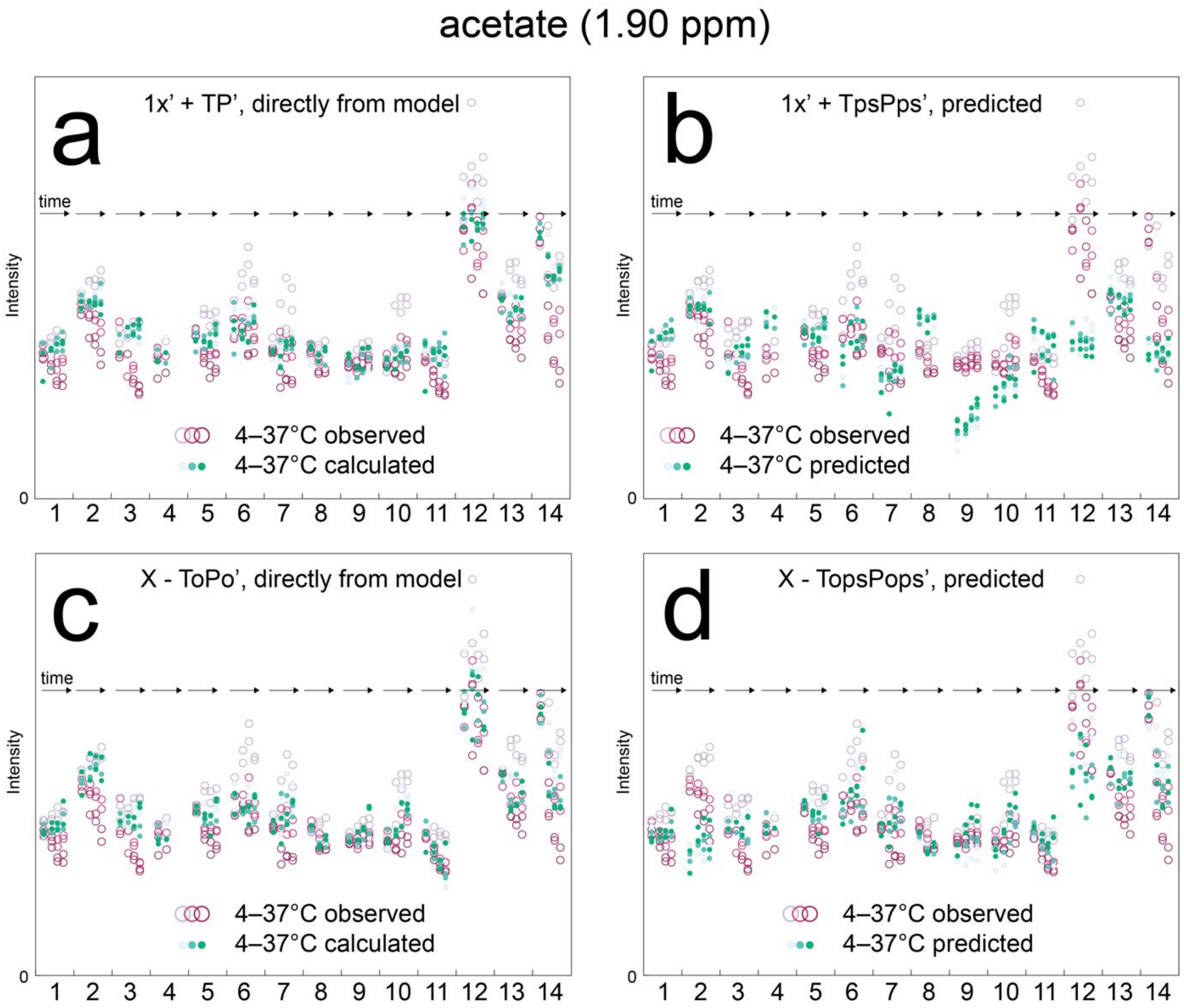

Supplemental figure 6. Acetate concentrations calculated directly from **a)** the components and **c)** orthogonal components of the original model, as well as predicted from **b)** the components and **d)** orthogonal components of corresponding model where the participant was excluded.

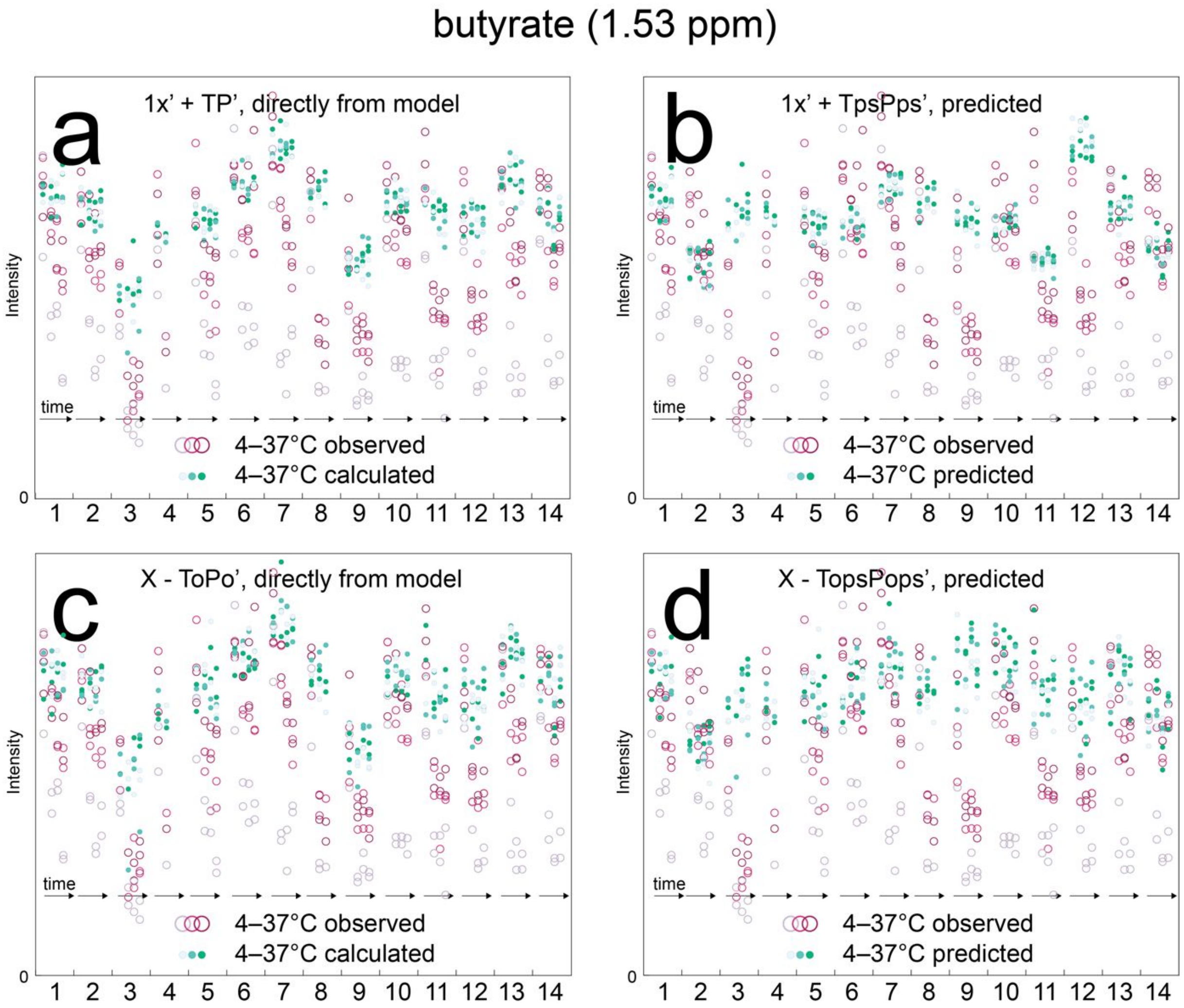

Supplemental figure 7. Butyrate concentrations calculated directly from **a)** the components and **c)** orthogonal components of the original model, as well as predicted from **b)** the components and **d)** orthogonal components of corresponding model where the participant was excluded.

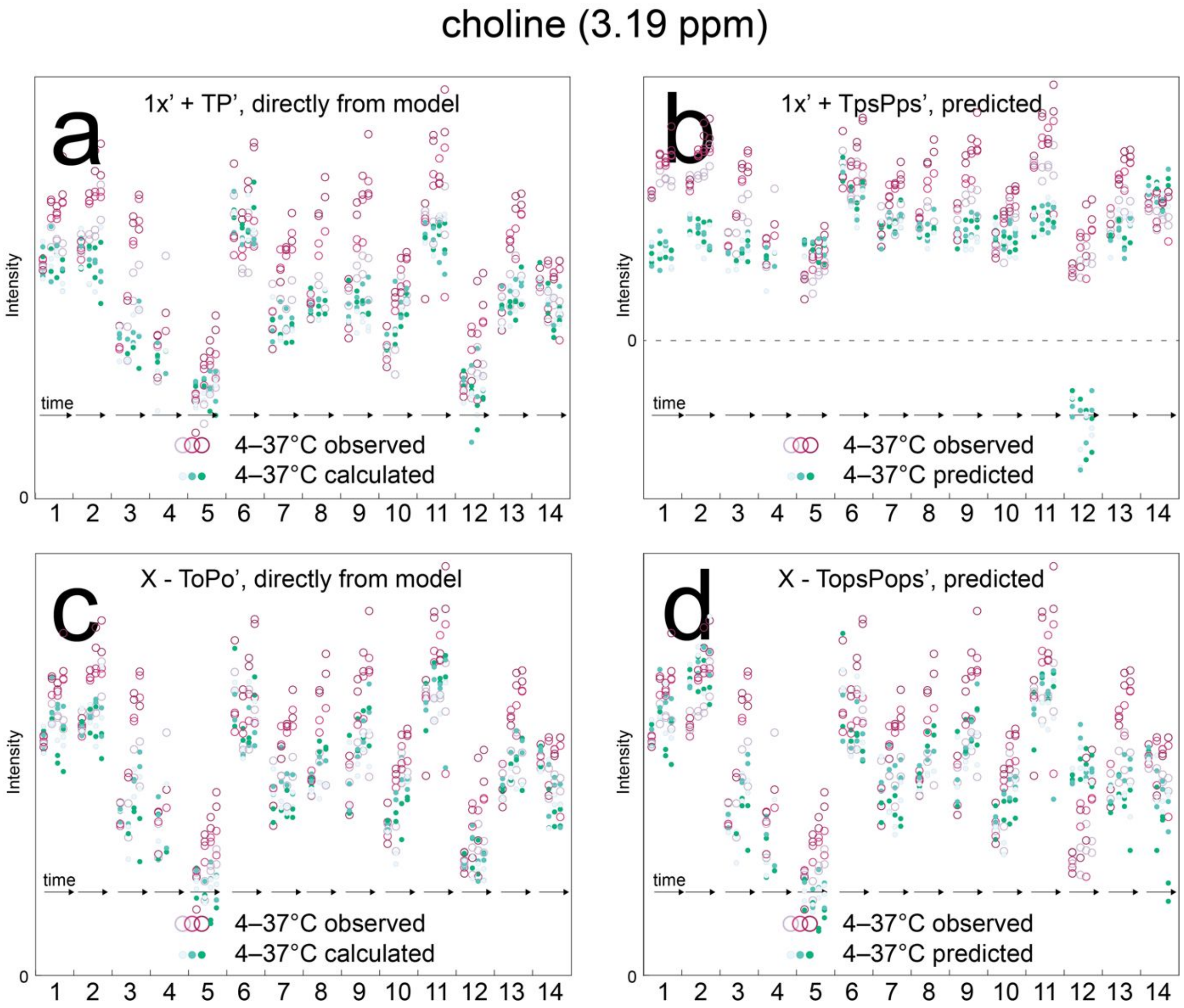

Supplemental figure 8. Choline concentrations calculated directly from **a)** the components and **c)** orthogonal components of the original model, as well as predicted from **b)** the components and **d)** orthogonal components of corresponding model where the participant was excluded.

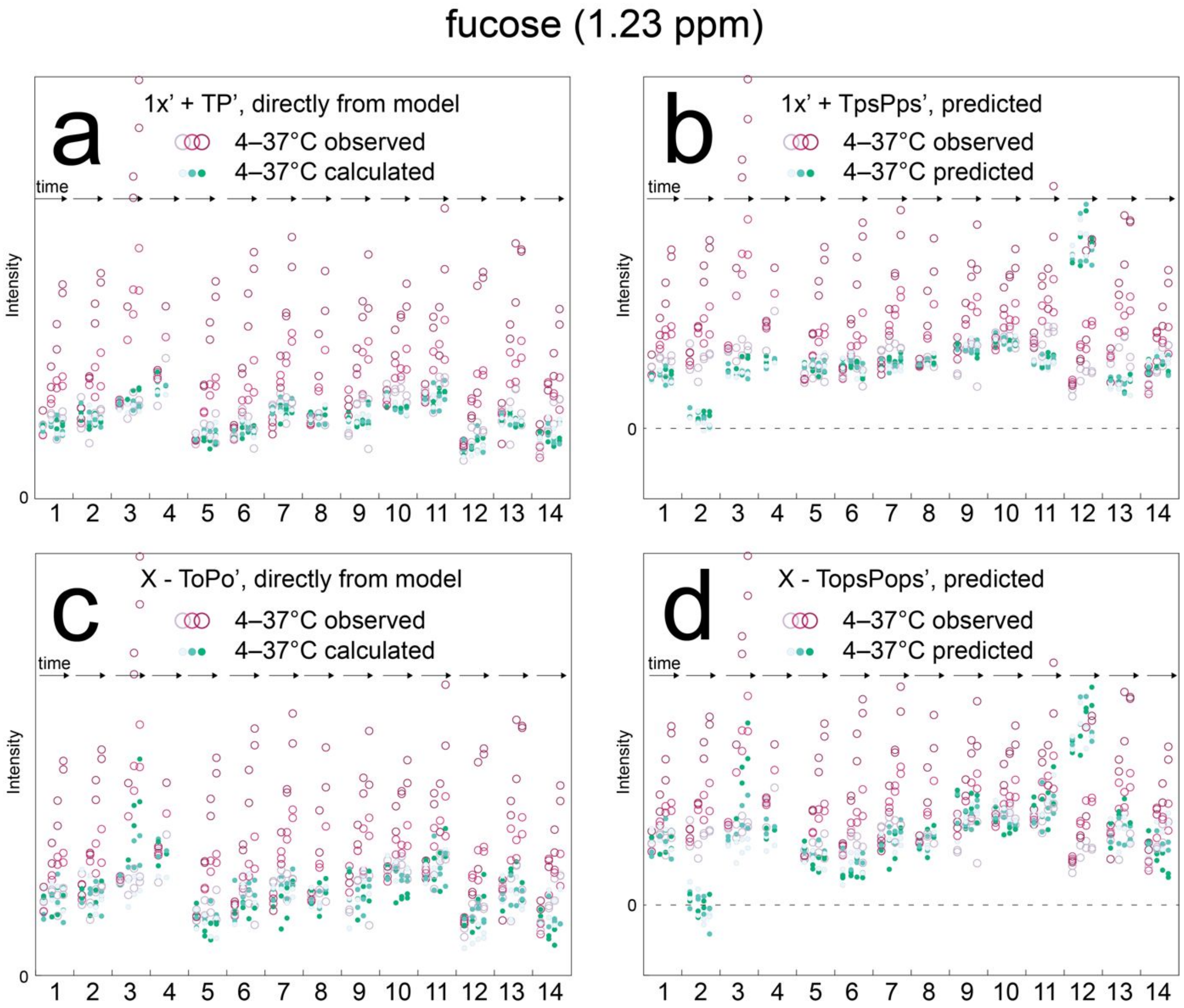

Supplemental figure 9. Fucose concentrations calculated directly from **a)** the components and **c)** orthogonal components of the original model, as well as predicted from **b)** the components and **d)** orthogonal components of corresponding model where the participant was excluded.

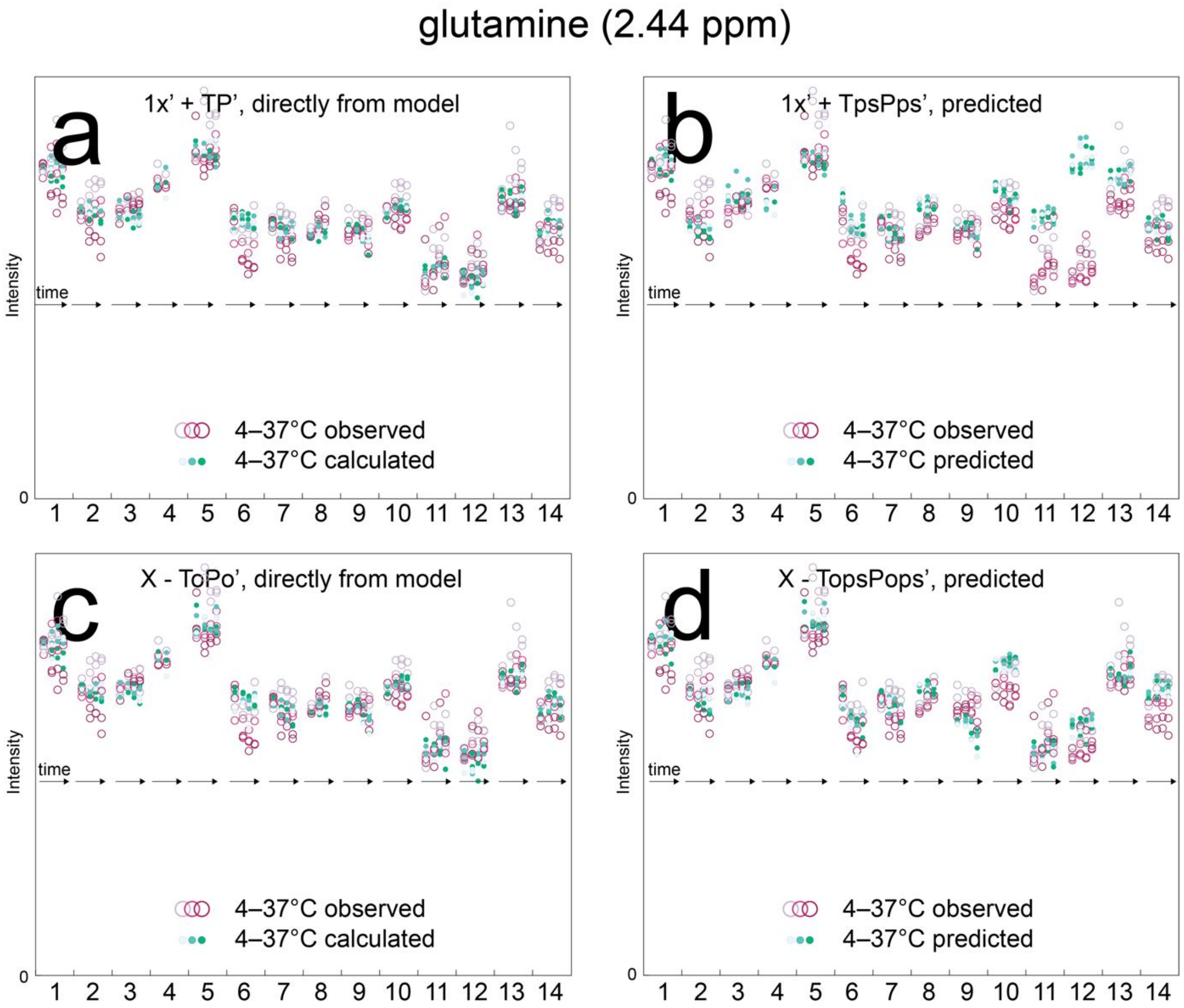

Supplemental figure 10. Glutamine concentrations calculated directly from **a)** the components and **c)** orthogonal components of the original model, as well as predicted from **b)** the components and **d)** orthogonal

components of corresponding model where the participant was excluded.

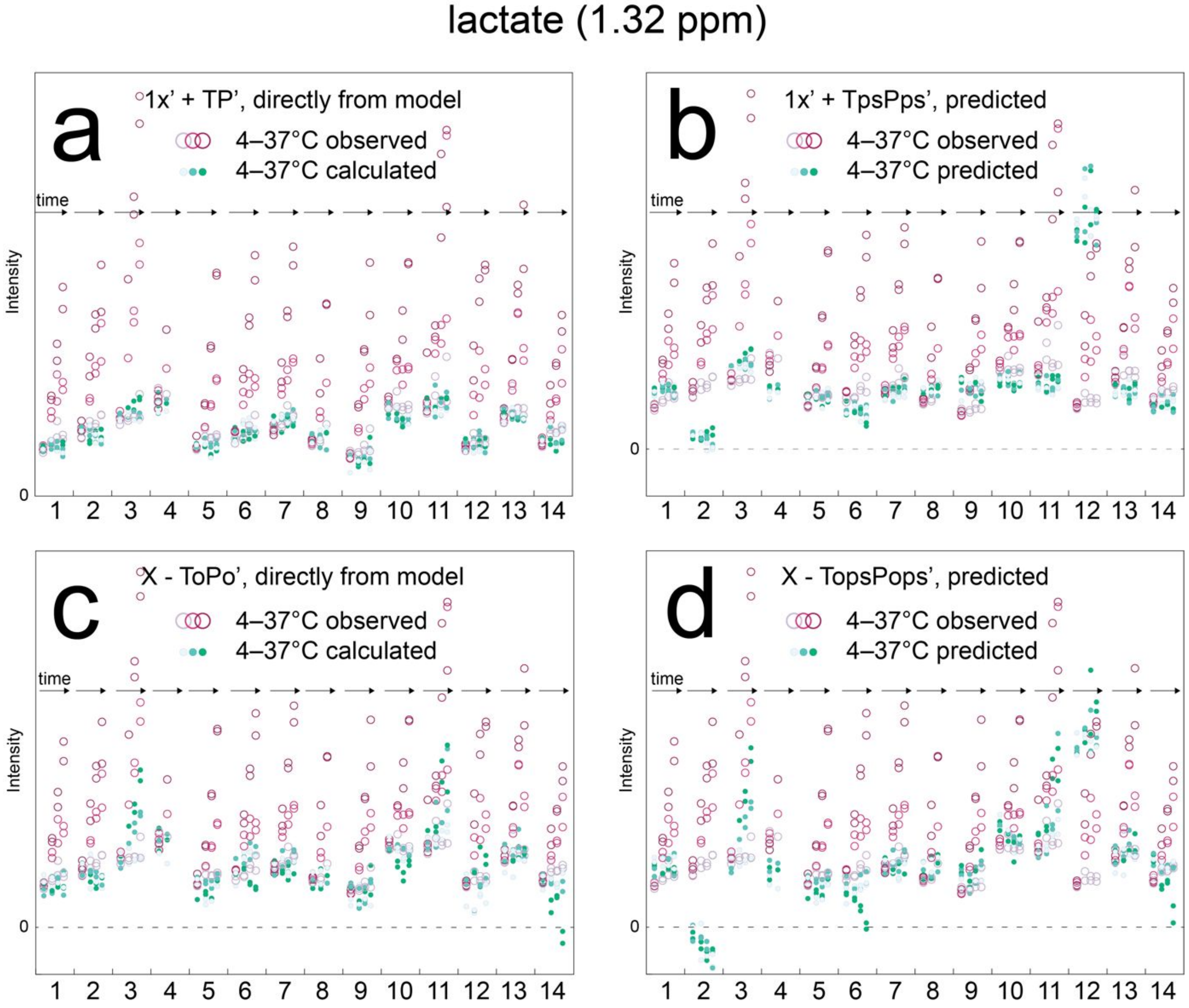

Supplemental figure 11. Lactate concentrations calculated directly from **a)** the components and **c)** orthogonal components of the original model, as well as predicted from **b)** the components and **d)** orthogonal components of corresponding model where the participant was excluded.

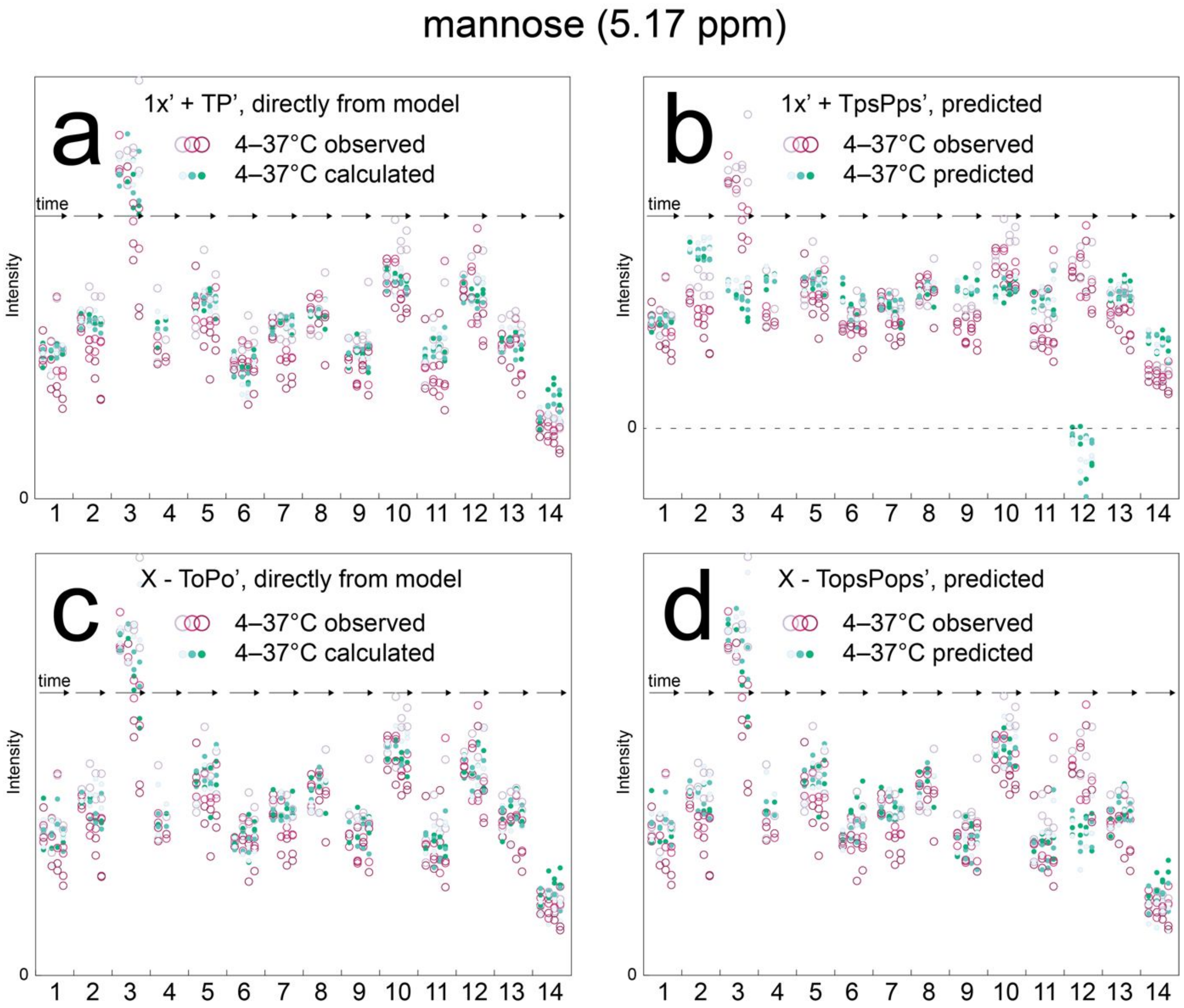

Supplemental figure 12. Mannose concentrations calculated directly from **a)** the components and **c)** orthogonal components of the original model, as well as predicted from **b)** the components and **d)** orthogonal components of corresponding model where the participant was excluded.

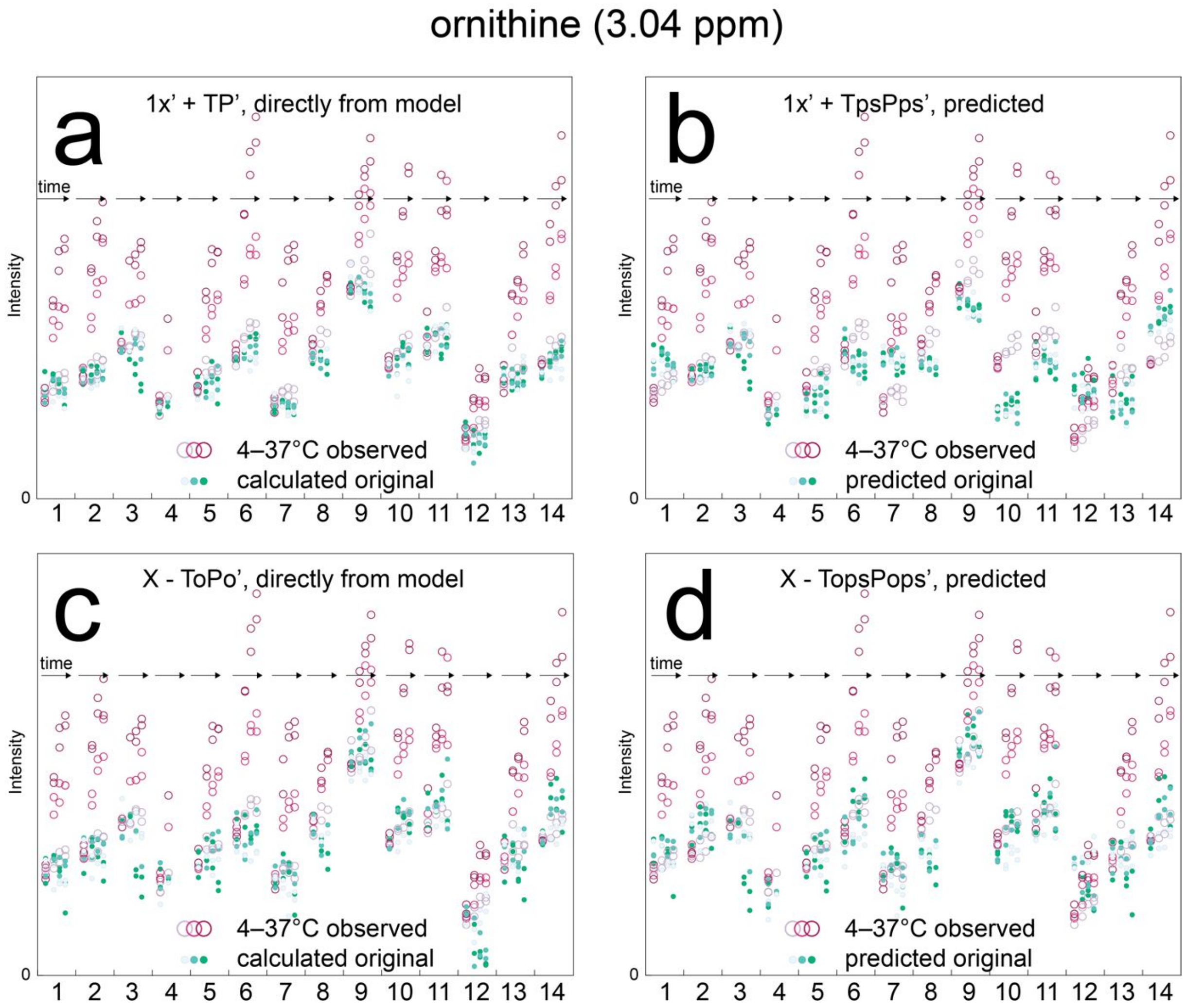

Supplemental figure 13. Ornithine concentrations calculated directly from **a)** the components and **c)** orthogonal components of the original model, as well as predicted from **b)** the components and **d)** orthogonal components of corresponding model where the participant was excluded.

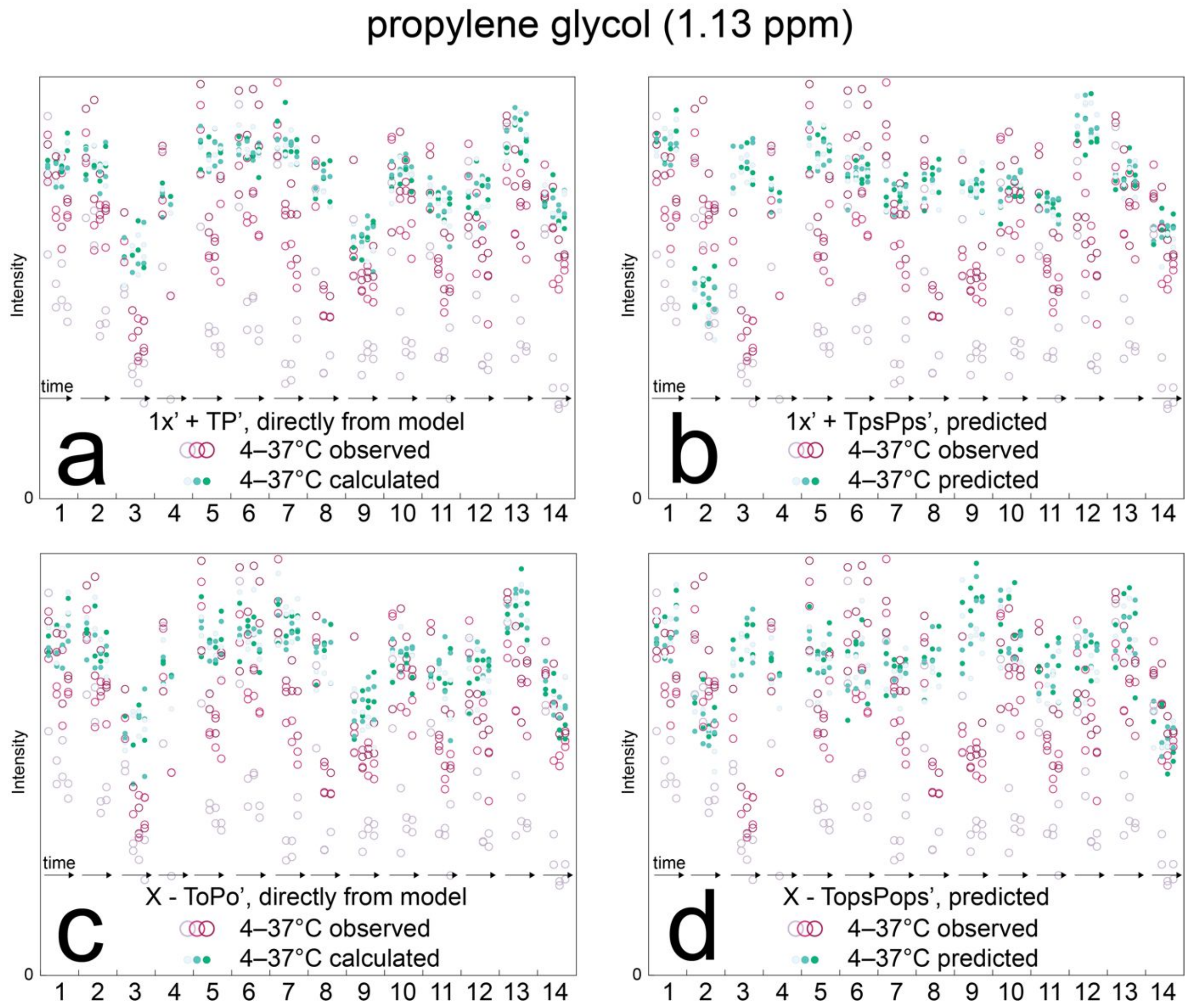

Supplemental figure 14. Propylene glycol concentrations calculated directly from **a)** the components and **c)** orthogonal components of the original model, as well as predicted from **b)** the components and **d)** orthogonal components of corresponding model where the participant was excluded.

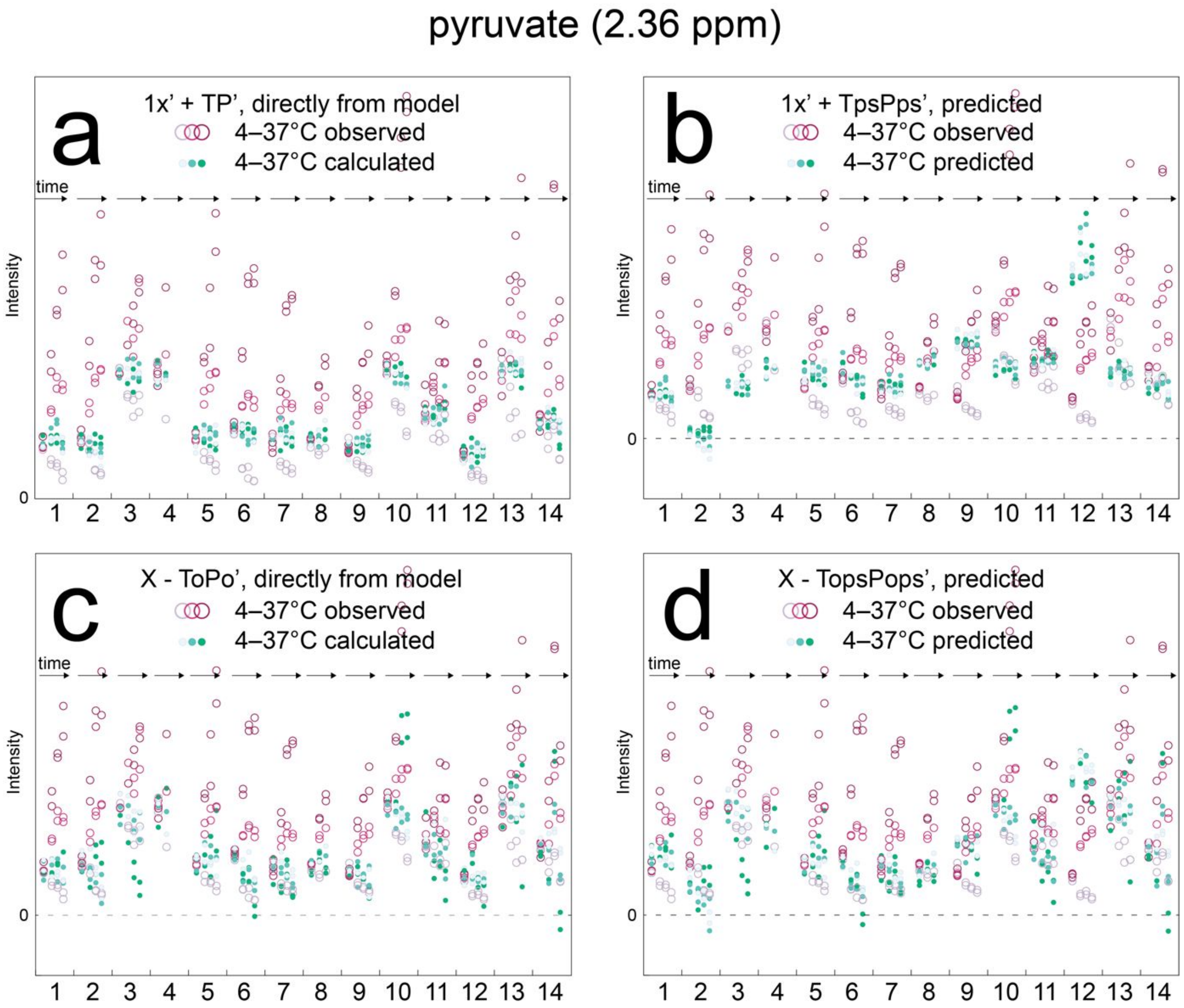

Supplemental figure 15. Pyruvate concentrations calculated directly from **a)** the components and **c)** orthogonal components of the original model, as well as predicted from **b)** the components and **d)** orthogonal components of corresponding model where the participant was excluded.

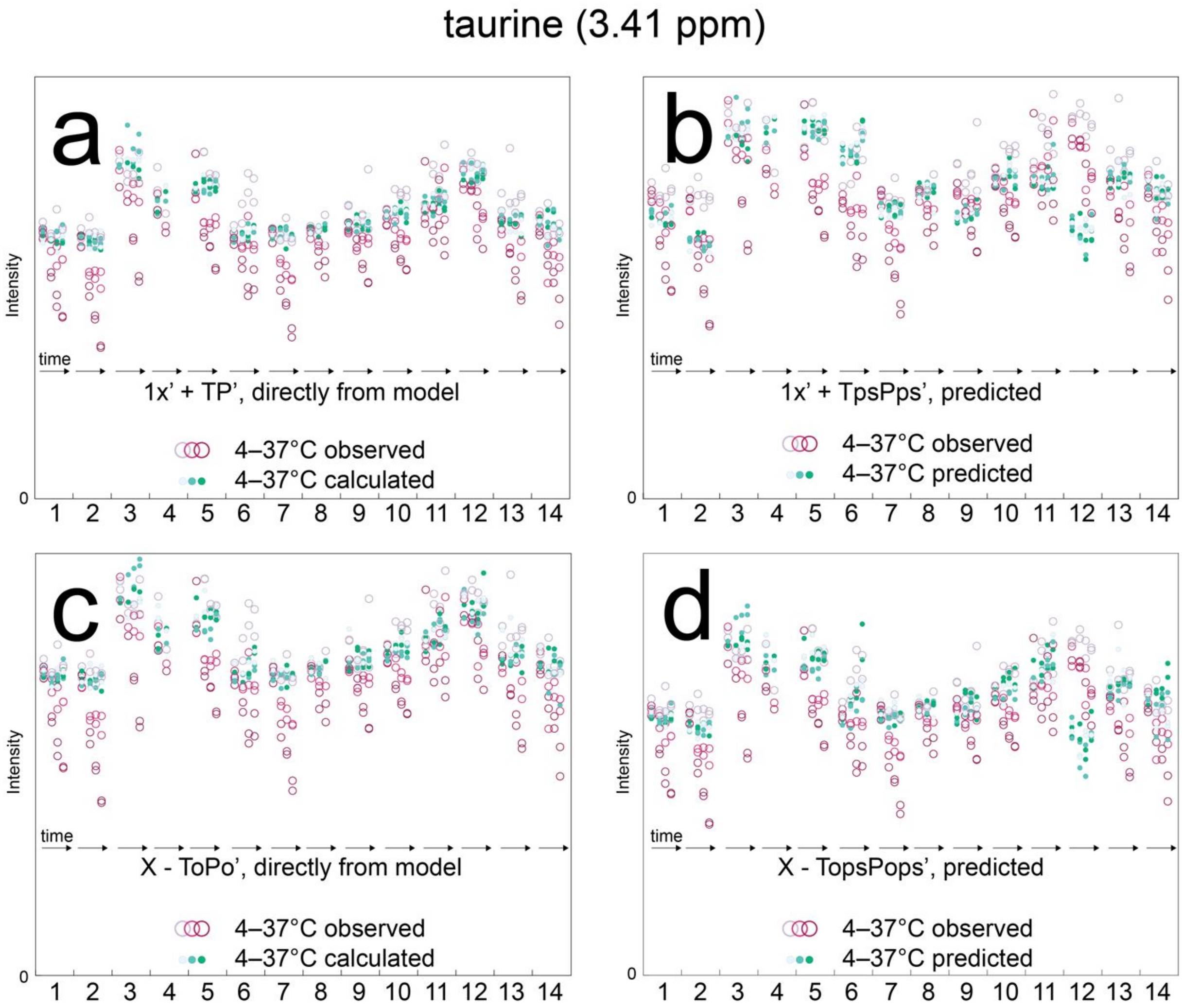

Supplemental figure 16. Taurine concentrations calculated directly from **a)** the components and **c)** orthogonal components of the original model, as well as predicted from **b)** the components and **d)** orthogonal components of corresponding model where the participant was excluded.

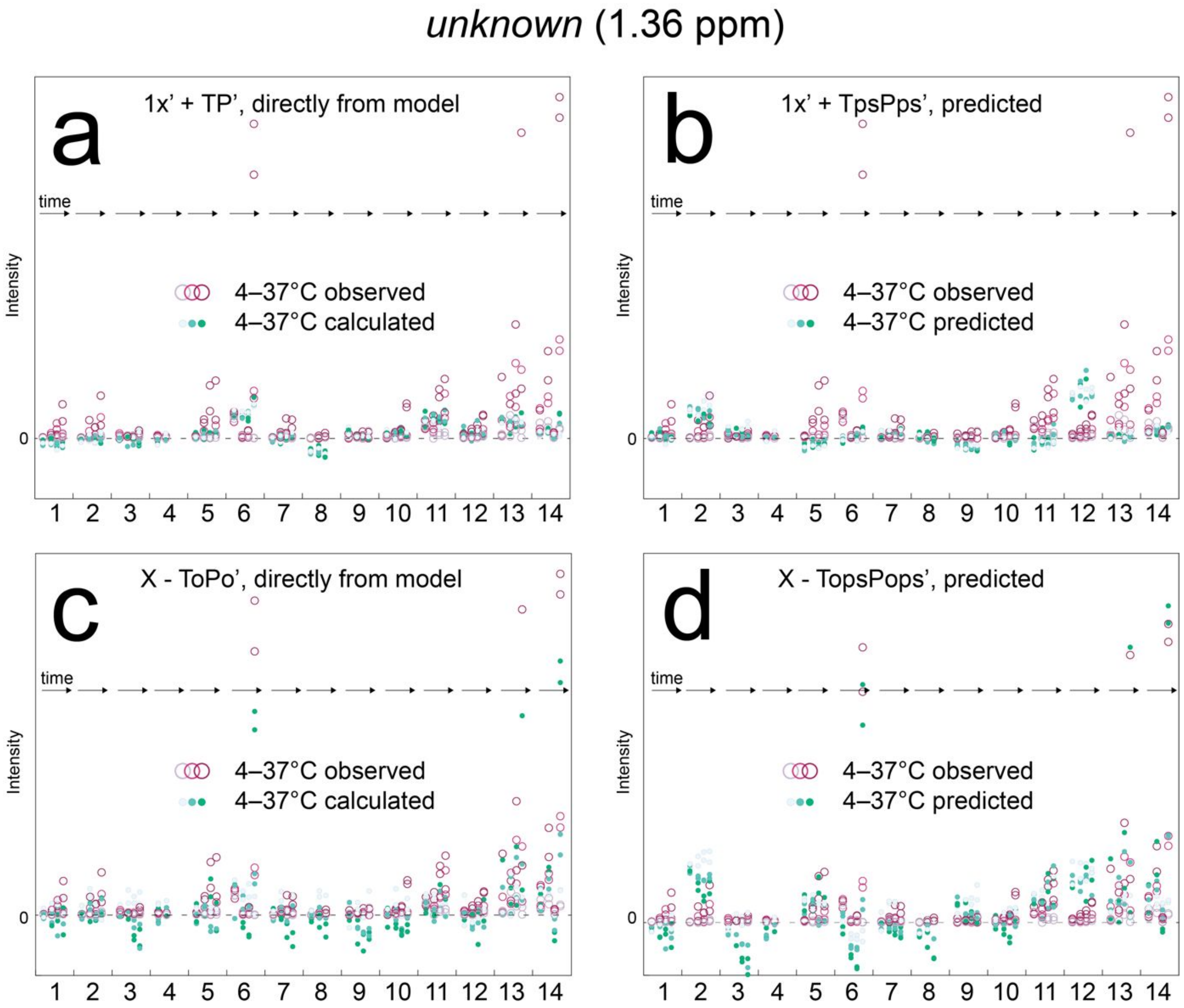

Supplemental figure 17. 1.36ppm unknown peak concentrations calculated directly from **a)** the components and **c)** orthogonal components of the original model, as well as predicted from **b)** the components and **d)** orthogonal components of corresponding model where the participant was excluded.

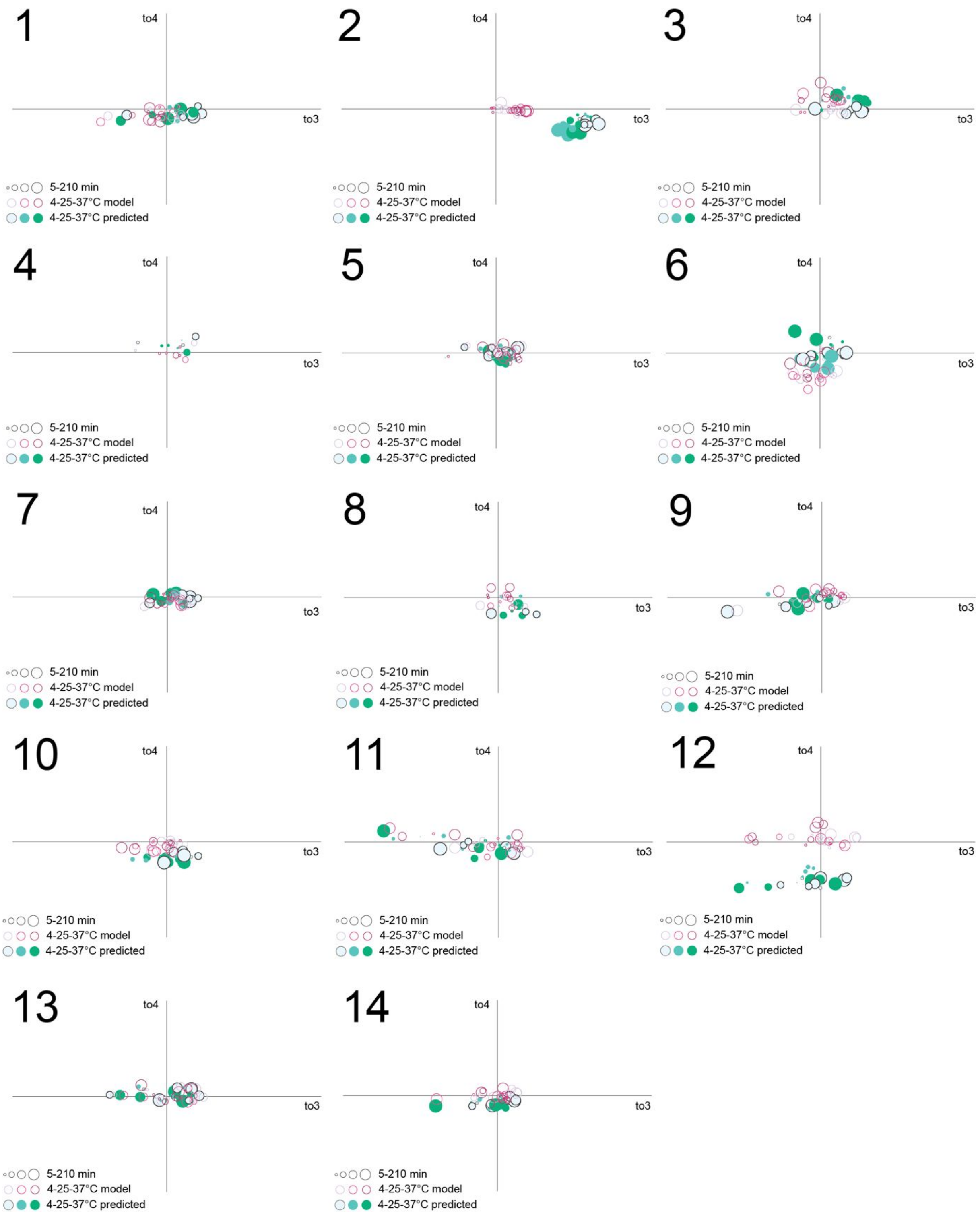

Supplemental figure 18. Illustrating the same as Figure 5 but 3rd vs 4th orthogonal scores rather than 1st vs 2nd, R^2X values ranging from 0.0429 to 0.0457, and from 0.00644 to 0.00793. While the first two orthogonal scores showed clear correspondence with induced incubation changes, the 3rd and 4th orthogonal scores show less obvious trends. The 3rd component describes minor dilution differences followed by normalization (not shown). The absolute numbers of these orthogonal scores are smaller compared to the first two and their impact when calculating metabolite concentrations is generally smaller. As for the first two orthogonal scores, the participants with high distance to model and Hotelling's T2, *e.g.* participant 12 and 2, tend to have a calculation vs. prediction bias.

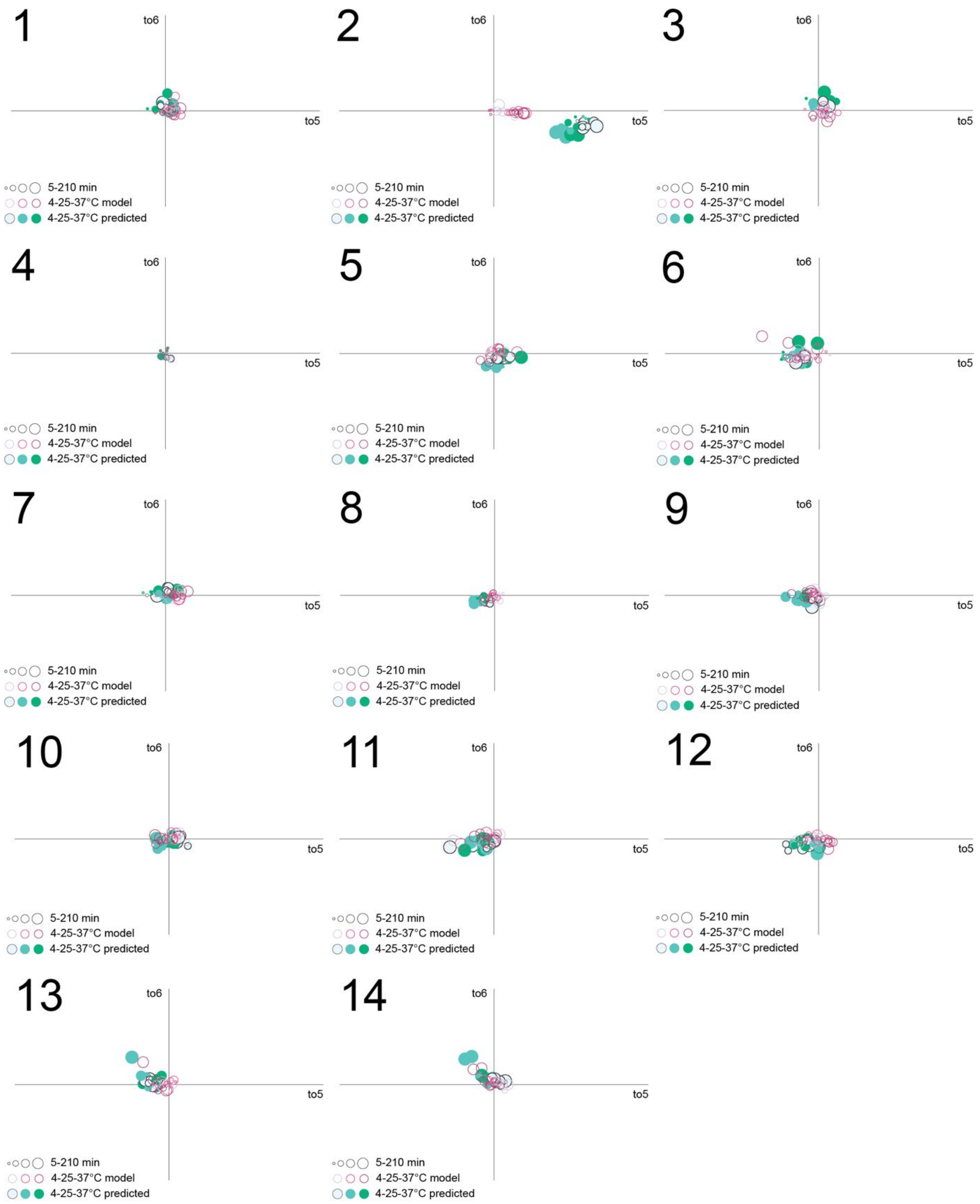

Supplemental figure 19. Illustrating the same as Figure 5 and Supplemental figure 18 but for the 5th vs 6th orthogonal scores, R^2X values ranging from 0.00560 to 0.00720, and from 0.00541 to 0.00576. The advantage of using more than four orthogonal components can be questioned. In our data it efficiently predicted almost zero intensity for an unknown molecule which starting from almost nothing increased with time in a subset of samples but not in others (not shown). Apart from that, in this case we wanted to demonstrate that more orthogonal components can be used which potentially could take care of incubation changes not observed in our small sample set.

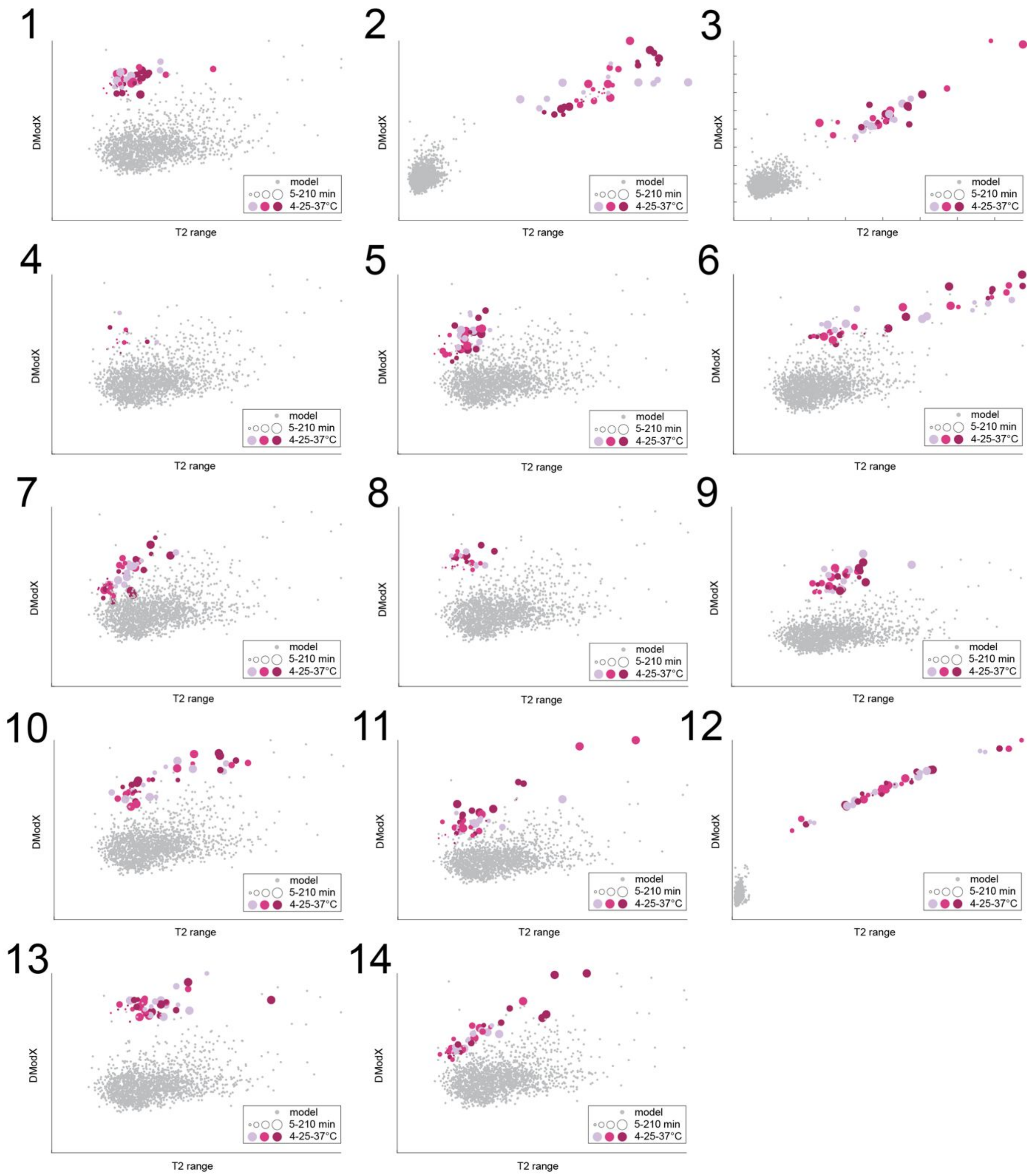

Supplemental figure 20. Distance to model (DModX) vs Hotelling's T2 range for 14 predicted participants. Prediction modelling performed generally well estimating under which conditions and incubation times samples had experienced, and could also for some metabolites sensitive to the incubation predict useful corresponding original values. But three participants (2, 3 and 12) with large distances to the model plane and Hotelling's T2 values were not predicted well with large biases showing that predictions are only useful if the predicted sample resembles at least some other in the model.
